# Design and Validation of Elastic Dies for Enhanced Metal Powder Compaction: A FEM and Experimental Study

**DOI:** 10.3390/ma18194491

**Published:** 2025-09-26

**Authors:** Dan Cristian Noveanu, Simona Noveanu

**Affiliations:** 1 Materials Science and Engineering Department, Technical University of Cluj-Napoca, 400641 Cluj-Napoca, Romania; dan.noveanu@ipm.utcluj.ro; 2 European Union, European University of Technology EUt+; 3 Mechatronics and Machine Dynamics Department, Technical University of Cluj-Napoca, 400641 Cluj-Napoca, Romania

**Keywords:** elastic sleeve, ejection force, finite element modeling, powder metallurgy, relative density, shrink-fit die, taper angle, viscoplastic model

## Abstract

**Highlights:**

**What are the main findings?**

- Elastic-sleeve dies enhance densification in powder compaction. Optimal taper angle α ≈ 3° maximizes contact pressure and density.
- Axial pretension Δh = 1.0–1.5 mm improves uniformity without overstress.

**What is the implication of the main finding?**

- FEM–experiment agreement within ±5% validates the constitutive model.
- Elastic dies reduce ejection defects compared to conventional rigid dies.

**Abstract:**

Metal powder compaction in rigid dies often suffers from high ejection forces, non-uniform density, and accelerated tool wear. We investigate an elastic-sleeve die concept in which a conical shrink-fit sleeve provides controllable radial confinement during pressing and elastic relaxation during extraction. An extensive experimental program on Fe-based and 316L powders, carried out in parallel with finite element analyses (SolidWorks Simulation version 2021; Marc Mentat 2005), quantified the roles of taper angle (α = 1–4°), axial pretension (Δh = 0.5–1.5 mm), and friction. Contact pressure increased from ≈52 MPa at α = 1° to ≈200 MPa at α = 3°, with negligible gains beyond 3°. For 316L, relative density reached ρ ≈ 0.889 at 325 kN with Δh = 1.5 mm; Fe–Cu–C achieved ρ ≈ 0.865 under identical conditions. The experimental results provided direct validation of the FEM, with calibrated viscoplastic simulations reproducing density–force trends within ≈±5% (mean density error ≈ 4.6%), while mid-stroke force differences (≈15–20%) reflected rearrangement/friction effects not captured by the constitutive law. The combined evidence identifies an optimal window of α ≈ 3° and Δh ≈ 1.0–1.5 mm that maximizes contact pressure and densification without overstressing the sleeve. Elastic relaxation of the sleeve facilitates extraction and suggests reduced ejection effort compared with rigid dies. These findings support elastic dies as a practical route to improved densification and tool life in powder metallurgy.

## 1. Introduction

Achieving metallic powder parts with densities close to the theoretical value requires exceptionally high compaction pressures and robust equipment, with significant capital cost. While higher density is essential for superior mechanical properties, it also intensifies extraction challenges: ejection forces rise with relative density, cracks and delamination can occur, and die wear accelerates; beyond certain density thresholds, these defects may appear irrespective of lubrication. These limitations motivate alternative compaction approaches capable of reaching very high relative densities (≈99.5%) while reducing extraction-related damage.

Powder metallurgy (PM) combines powder production, dosing, compaction, and sintering to deliver near-net-shape parts with high material utilization, typically exceeding 97% [1,2,3]. Compaction is commonly performed by applying vertical pressure with lateral confinement from die walls, and the required pressure depends on powder distribution, lateral flow, and tolerance targets [4]. Process variants (single- or double-action) are selected by geometry/size: single-action for thin, uniform sections [5,6]. Yet, due to wall friction, uniaxial pressing induces non-uniform density fields, especially near the base, as documented in classical analyses [4]. Double-action pressing improves symmetry and permits taller compacts [7], though density remains governed by shape and size, with characteristic neutral-axis symmetry and persistent gradients [8,9].

Beyond conventional rigid dies, elastic sleeve dies offer controlled radial confinement during pressing and elastic relaxation during extraction, reducing ejection forces, limiting density gradients, and extending tool life [10,11,12,13,14]. Prior works describe the elastic-sleeve concept for powder compaction to avoid taper defects and cracks while reducing wear on active surfaces [14,15,16,17,18,19]. Because radial pressure amplifies die-wall friction and ejection force [20,21], and spring-back affects dimensional stability [22,23], numerous studies have explored ways to reduce green compacts–die friction via lubrication strategies and surface treatments [24,25,26,27,28,29].

Modeling of powder-compaction processes has a substantial background [30,31,32,33,34,35] and is now greatly strengthened by advanced computation and software. In this work, we combine finite element analyses with experiments to quantify how geometry and contact parameters govern stress transfer and densification in elastic dies. We evaluate taper angle (α), axial pretension (Δh), and interfacial friction (μ) on contact pressure, sleeve stresses, and compaction response, and validate the findings on Fe-based and stainless-steel powders. Unlike studies that vary only one factor at a time, this investigation explicitly couples α, Δh, and μ in a unified FEM–experiment framework, thereby providing quantitative design guidance for elastic-die powder compaction.

Finite element modeling has been extensively applied to powder compaction using constitutive laws that account for pressure sensitivity and density dependence. Classical models such as Drucker–Prager Cap (DPC) and Cam–Clay capture densification but often require extensive calibration [36,37]. Alternative approaches, including the Shima–Oyane yield function and viscoplastic formulations, have been employed for stainless steel powders to describe relative density evolution under uniaxial and isostatic pressing [38,39]. Despite these advances, simulations frequently underpredict force in the intermediate stroke region due to particle rearrangement and evolving wall friction, phenomena that are difficult to represent in continuum models. The present study builds on this background by explicitly coupling taper angle (α), axial pretension (Δh), and interfacial friction (μ) in a unified FEM–experiment framework, thereby providing quantitative design guidance for elastic-die powder compaction.

## 2. Materials and Methods

### 2.1. The Functional Principle of the Elastic Die

**Figure 1:**
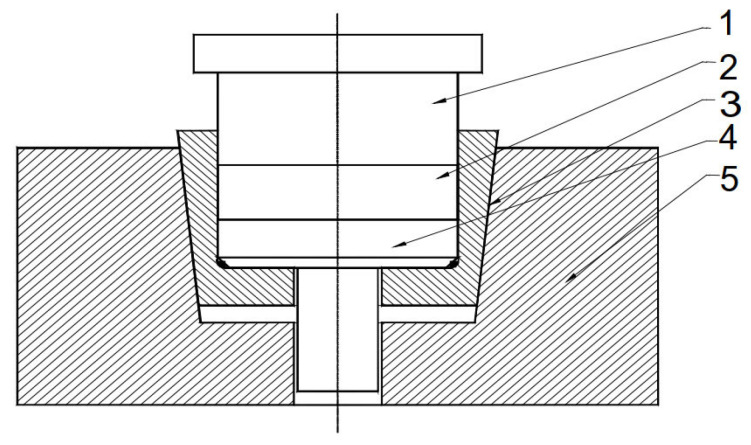
The principle of the elastic die: 1—Upper punch; 2—Compact; 3—Elastic sleeve; 4—Extractor; 5—Die support.

The elastic die concept introduces a shrink-fitted conical sleeve that undergoes radial contraction during pressing and elastic relaxation during extraction—Figure 1 [15].

The applied pressing force induces triaxial stresses within the elastic die, causing radial expansion and a corresponding transverse contraction in the pressing direction. The performance of such dies depends on three design parameters: the taper angle (α), which governs the balance between radial confinement and axial slip; the axial pretension (Δh), which controls the initial radial shrinkage of the sleeve; and the interfacial friction coefficient (μ), which influences stress transfer and ejection behavior. These variables were selected as the focus of the numerical and experimental investigations presented in this work.

The elastic die concept was selected in this work because it provides several advantages compared with conventional rigid dies. The radial confinement during pressing and the subsequent elastic relaxation reduce ejection forces, limit density gradients, and minimize the risk of cracking in the green compact. In addition, the lower friction at the die–part interface extends tool life and enables higher densification at comparable pressing loads, making elastic dies a practical alternative for advanced powder compaction.

For completeness, additional theoretical derivations of the lateral-to-vertical pressure balance and early elastic-die schemes are provided in Appendix A.

### 2.2. Determination of the Optimal Taper Angle of the Elastic Sleeve

The following SolidWorks simulations are presented within the Methods as a preliminary design tool. Their purpose was to identify a feasible range of taper angles prior to the more detailed FEM analyses with Marc Mentat and the subsequent experimental validation. These results therefore form part of the methodology rather than the final results.

To identify the optimal taper angle of the elastic sleeve, a numerical calculation program was developed using the finite element method. To make these calculations, the SolidWorks Simulation finite element calculation code was used.

**Figure 2:**
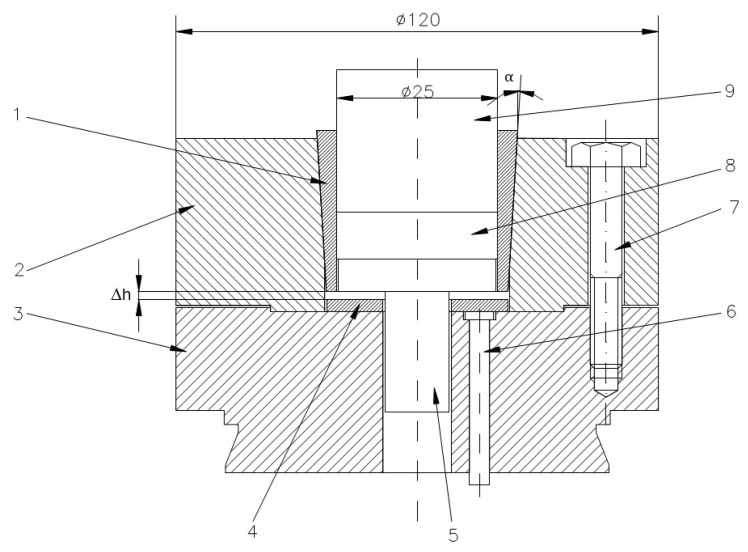
Tool system design for the cylindrical preforms compaction: 1—elastic sleeve, 2—upper die support, 3—lower die support, 4—spacer ring, 5—base plate, 6—extractor rod, 7—assembly screw, 8—compacted powder, 9—punch. (the units are millimeters).

Figure 2 shows the tool system designed for producing cylindrical preforms using metal powder compaction in elastic dies.

**Figure 3:**
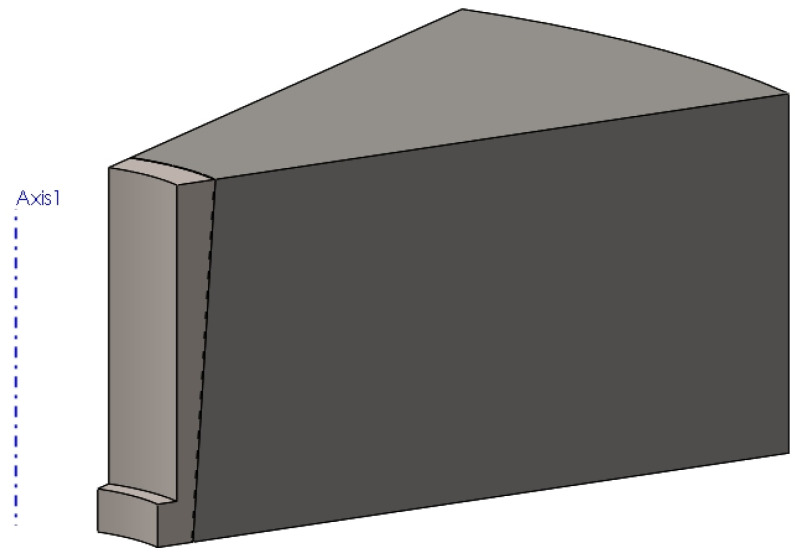
Elastic sleeve-upper die support assembly geometry used in numerical analysis.

The elastic sleeve is designed with a cylindrical inner bore, which defines the compaction cavity, and a conical outer surface that matches the conical slot of the upper die support. This shrink-fit geometry ensures intimate contact between the sleeve and the rigid support. During pressing, the taper angle (α) governs the balance between axial displacement and radial confinement: a smaller angle provides stronger radial pressure on the powder but increases frictional resistance during ejection, while a larger angle facilitates extraction but reduces confinement. The conical interface therefore plays a central role in transmitting load from the press to the sleeve and controlling the stress state within the compact. After the powder compaction process has been completed, the elastic sleeve is depressed with the help of extractor rods. The elastic return of the sleeve and the relaxation of the compact take place simultaneously over their entire height, without the danger of cracks in the workpiece. Moreover, because the sleeve no longer tightens the compact, its extraction is done requiring a significantly reduced force. The sleeve was shrink-fitted with the upper die support by pressing axially downwards with different Δh. By using three spacer rings of different heights, three axial press depths of the sleeve were obtained, namely: Δh = 0.5 mm, 1 mm and 1.5 mm. From the assembly presented in Figure 2, only the elastic sleeve and the die support were included in this study. Given that the assembly is axially symmetrical, to reduce the calculation time of the application, the numerical analysis was performed on a sector of 30° of the circumference, shown in Figure 3.

To conduct the analysis aimed at determining the size of the discretization network, a network composed of tetrahedral elements was selected.

The values of the friction coefficient introduced in the simulation procedure vary in the range of 0.05–0.15, close to those encountered in the practice of designing such plastic deformation tools.

Also, four taper angles of the sleeve were considered in these FEM simulations: α = 1°, 2°, 3° and 4°.

**Figure 4:**
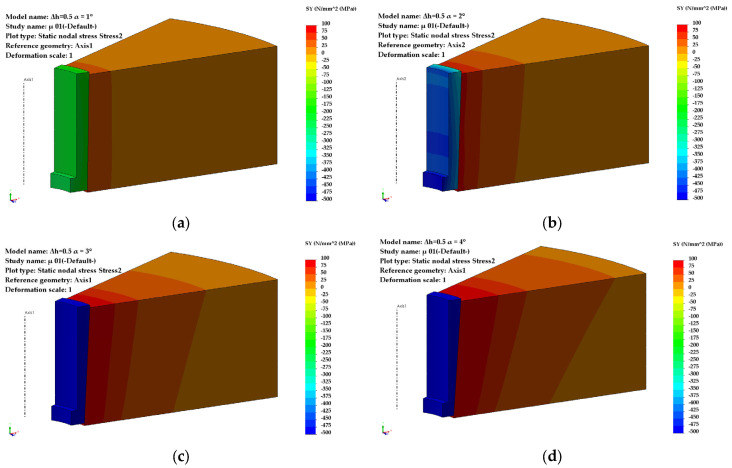
Distribution of circumferential stresses (Δh = 0.5 mm): (**a**) α = 1°, (**b**) α = 2°, (**c**) α = 3°, (**d**) α = 4°.

**Figure 5:**
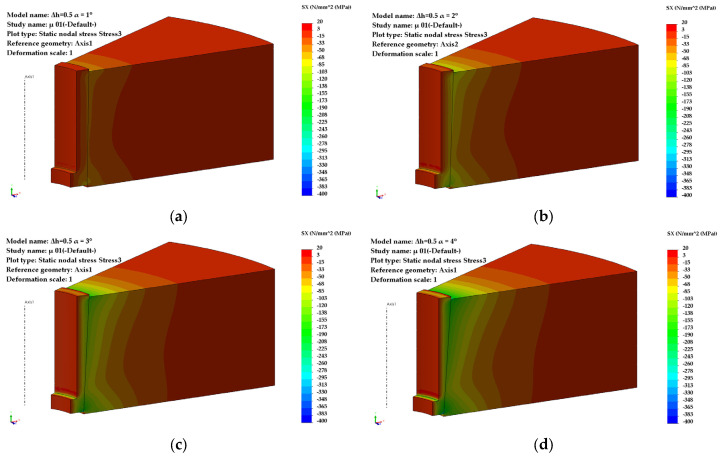
Distribution of radial stresses (Δh = 0.5 mm): (**a**) α = 1°, (**b**) α = 2°, (**c**) α = 3°, (**d**) α = 4°.

For a pre-compression depth of the sleeve, Δh = 0.5 mm, the circumferential stresses (Figure 4) and radial stresses (Figure 5) evolve over a wide range of values, depending on the inclination angle and the coefficient of friction. The direction of action of the circumferential stresses is compressive, and their values vary from −115 MPa (α = 1°) to −576 MPa (α = 4°).

**Figure 6:**
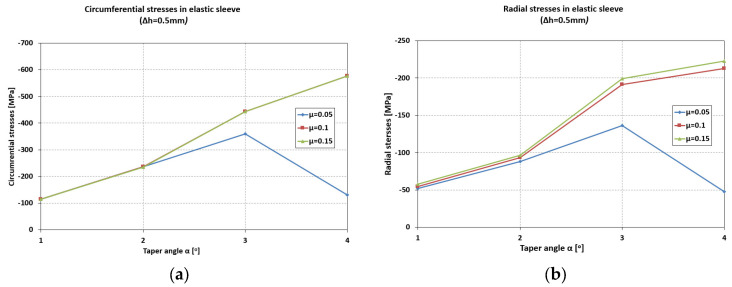
Maximum values of circumferential (**a**) and radial (**b**) stresses in the elastic sleeve.

The influence of the coefficient of friction becomes noticeable at taper angles greater than 2°50′ (Figure 6). For low values of the friction coefficient (μ = 0.05), a maximum appears in the evolution of the circumferential stresses, occurring at an angle α = 2°50′, with a value of 370 MPa.

For higher values of the friction coefficient (μ = 0.1–0.15), the increasing trend of the circumferential stresses continues even at larger taper angles (α = 4°).

The radial stresses (Figure 6) are compressive, ranging from −51 MPa (α = 1°) to −220 MPa (α = 4°) for μ = 0.1–0.15. For μ = 0.05, the values of the radial stresses in the sleeve are significantly lower at large angles (−49 MPa, α = 4°) compared to small ones.

**Figure 7:**
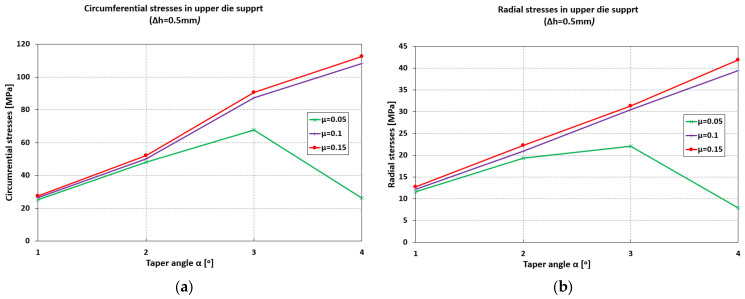
Maximum values of circumferential (**a**) and radial (**b**) stresses in the upper die support.

In the upper die support, both circumferential and radial stresses are tensile (Figure 7). The influence of friction is significant for high friction coefficients (μ = 0.1–0.15), with an almost linear increasing trend: 28 MPa (α = 1°) to 110 MPa (α = 4°) for circumferential stresses, 15 MPa (α = 1°) to 40 MPa (α = 4°) for radial stresses.

**Figure 8:**
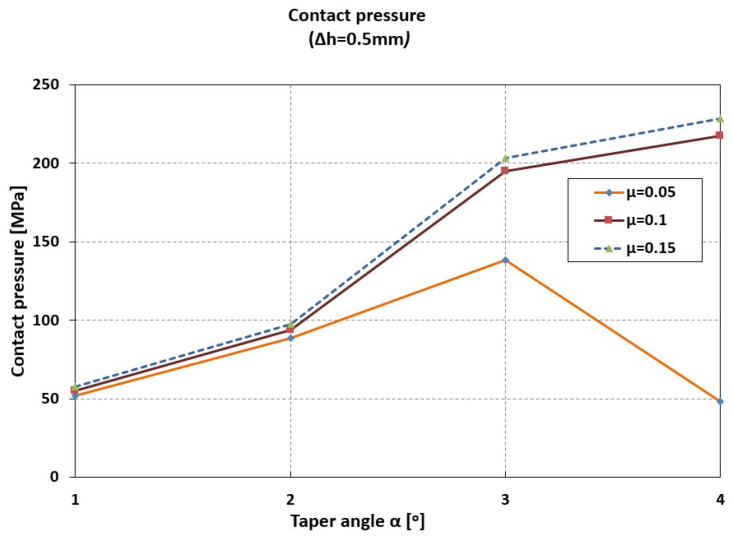
Maximum values of contact pressure.

The contact pressure increases by 85% (from 52 MPa to 96 MPa) when the angle increases from 1° to 2°, and by 300% (from 52 MPa to 200 MPa) when the angle increases from 1° to 3° (Figure 8).

An increase in taper angle from 3° to 4° results in only a 10% increase in contact pressure (μ = 0.1–0.15). At low friction coefficient values (μ = 0.05), the contact pressure decreases significantly at high taper angles (140 MPa at 3°, and 49 MPa at 4°).

**Figure A3:**
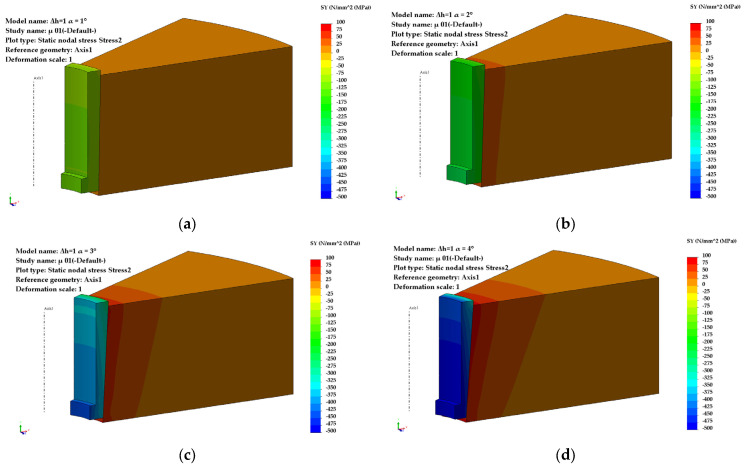
Distribution of circumferential stresses (Δh = 1 mm): (**a**) α = 1°, (**b**) α = 2°, (**c**) α = 3°, (**d**) α = 4°.

**Figure A4:**
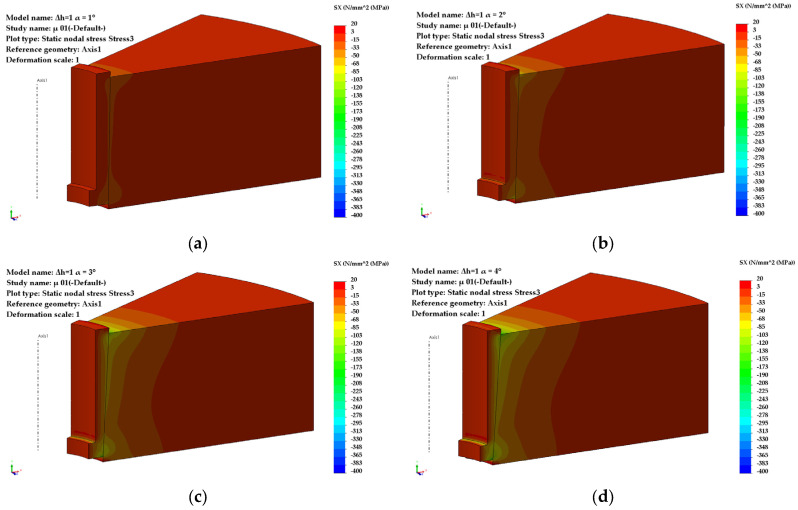
Distribution of radial stresses (Δh = 1 mm): (**a**) α = 1°, (**b**) α = 2°, (**c**) α = 3°, (**d**) α = 4°.

Pre-compression with Δh = 1 mm significantly increases the values of circumferential (Figure A3) and radial stresses (Figure A4). The direction of action of circumferential and radial stresses in the elastic sleeve remains compressive, and the trend of evolution is like that observed at 0.5 mm pre-compression. Given the deformation behavior within the elastic domain, a proportional increase in stress values was expected, but the multiplication ratio was of particular interest.

At a greater pre-compression depth, Δh = 1.5 mm, the radial stress becomes more pronounced, and a modification in the ratio between the two principal stresses—circumferential (Figure A3) and radial (Figure A4)—is observed, while maintaining the same direction of action and the same dimensional structure between the elastic sleeve and the container.

Data obtained for maximum values of circumferential stresses, stresses and contact pressures, both in the elastic sleeve and in the container, as well as their distribution, are presented in Appendix B.

**Figure 9:**
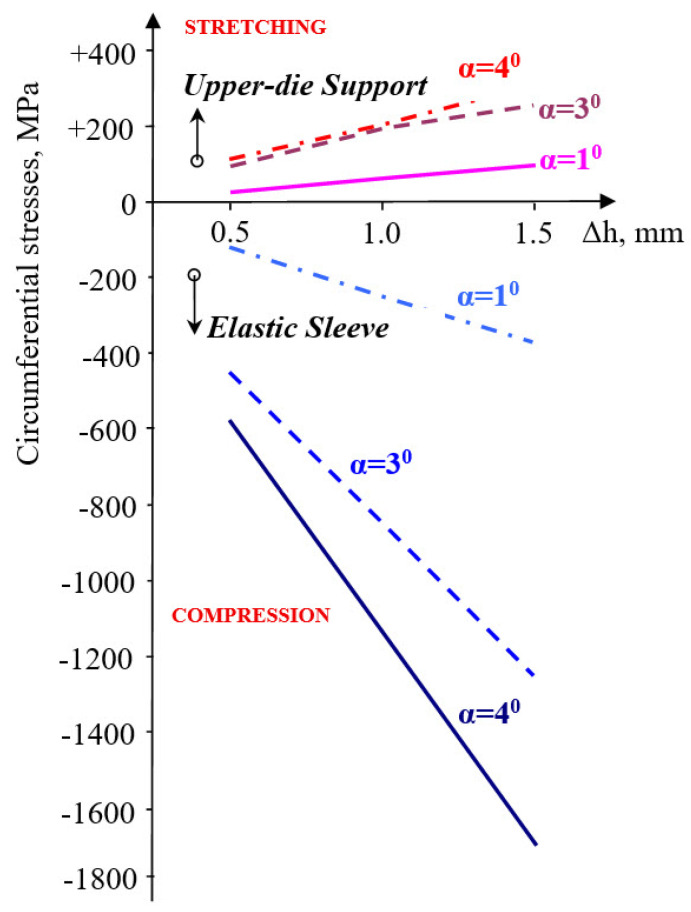
Variation in circumferential stresses in elastic sleeve and upper-die support.

With increasing the taper angle of the sleeve (container), the values of circumferential stresses (Figure 9) increase, in a linear dependence on the size of the precompression.

If for α = 1°, the circumferential stress in the elastic sleeve increases by 3.2 times when the pre-compression increases from Δh = 0.5 mm to Δh = 1.5 mm, at an angle of α = 3°, this increases by 1.82 times, and by 1.93 times at an angle equal to 4°. In the upper-die support, the increase is 2.42 times (α = 1°) and 1.68 times at an angle of 4°.

**Figure 10:**
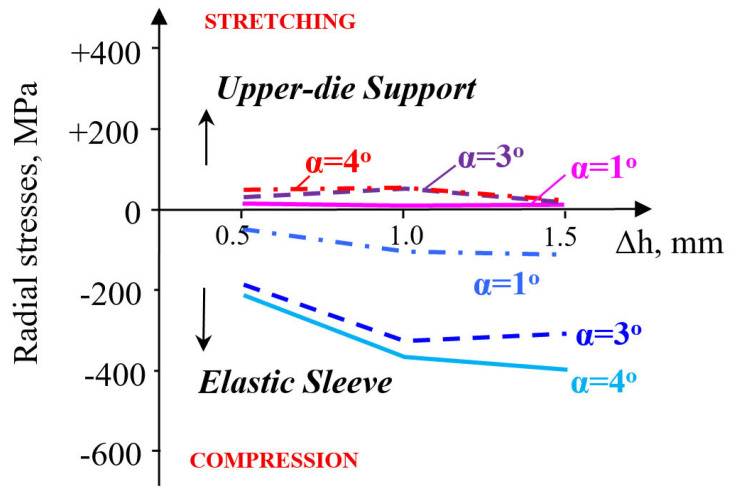
Variation in radial stresses in elastic sleeve and upper-die support.

The radial stresses are compressive in the sleeve (Figure 10), but a more significant evolution occurs at pre-compressions varying between 0.5 and 1 mm.

**Figure 11:**
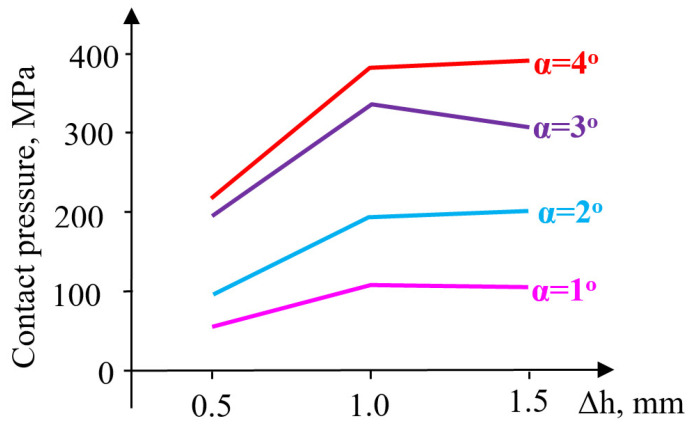
Variation in contact pressure.

A pre-compression Δh = 1–1.5 mm influences the radial stress very little. Moreover, its evolution is not sensitive to the increase in the taper angle. The radial stresses in the container are stretching but remain close for taper angles between 1 and 4° and are unaffected by the magnitude of pre-compression. The evolution of the contact pressure is influenced by the magnitude of pre-compression only in the interval Δh = 0.5–1 mm, increasingly so as the taper angle also increases (Figure 11).

Higher values of pre-compression influence the evolution of contact pressure insignificantly at small angles (α = 1–2°) and relatively little at α = 4°. At the taper angle α = 3°, the contact pressure decreases for pre-compressions between Δh = 1–1.5 mm. For a pre-compression value equal to Δh = 1 mm, the pressure increases from 107 MPa (α = 1°) to 334 MPa (α = 3°) and 379 MPa (α = 4°), showing an evolution proportional to the size of the angle. It is worth noting the favorable influence of increasing the angle up to 3°, a value beyond which a change occurs in the ratio between the two principal stresses, leading to a reduction in circumferential stresses and the inefficiency of elastic pre-compression.

**Figure 12:**
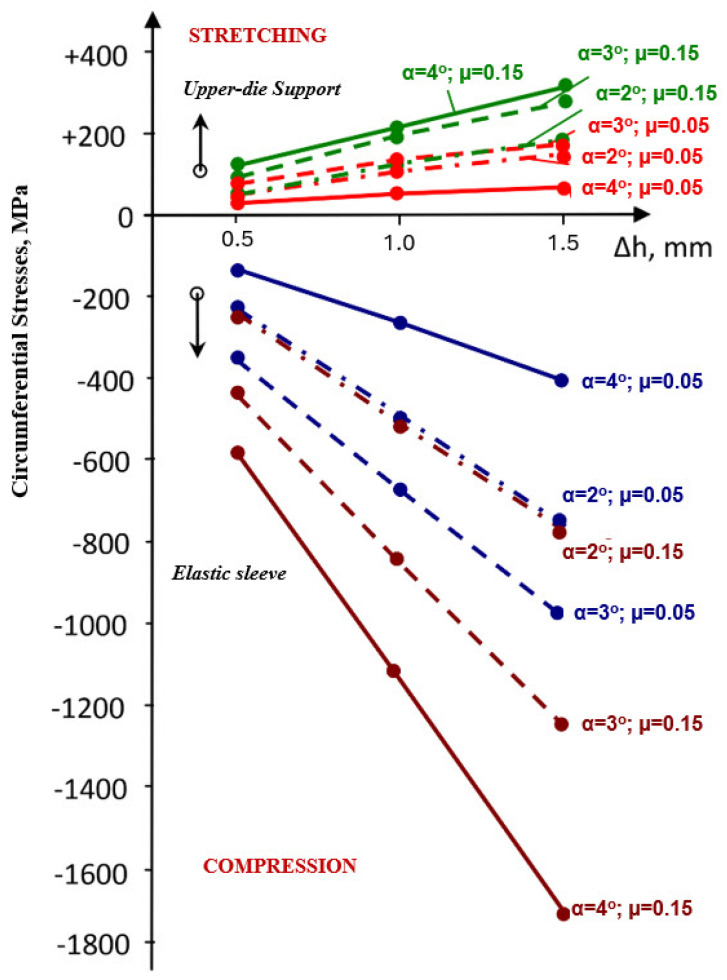
Evolution of circumferential stresses in the sleeve and in the upper-die body for different values of the taper angle α and friction coefficient μ.

Increasing the friction coefficient, in the range 0.05–0.15, causes a rise in the values of circumferential stresses both in the sleeve and in the container (Figure 12). Thus, in the sleeve, at a taper angle of 3°, an increase in the friction coefficient from 0.05 to 0.15 causes an increase in circumferential stresses by 1.2 times (for Δh = 0.5 mm) and by 1.3 times for a pre-compression Δh = 1.5 mm.

**Figure 13:**
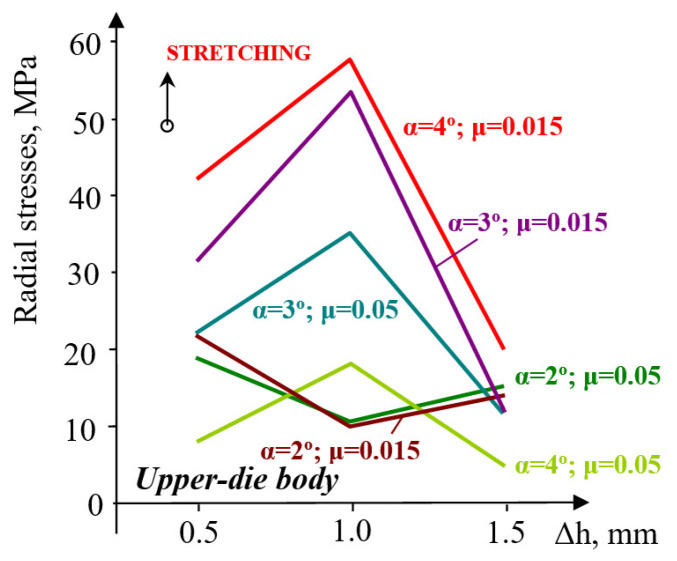
Evolution of radial stresses in the upper-die body for varying values of taper angle α and friction coefficient μ.

In the upper-die body, the increase in circumferential stress is smaller: approximately 1.1 times for the two extreme values of pre-compression considered. Increasing the taper of the sleeve (container) wall from 3° to 4° causes only a relatively small increase in stress. This means that raising the taper angle beyond 3° does not bring a significant improvement in the efficiency of the elastic structure. The evolution of radial stresses, both in the upper-die body and in the sleeve, is very different from that of circumferential stresses. In the upper-die body (Figure 13), the values of radial stresses corresponding to small angles decrease. This trend is noticeable at pre-compressions between 0.5 and 1.0 mm, regardless of the value of the friction coefficient.

**Figure 14:**
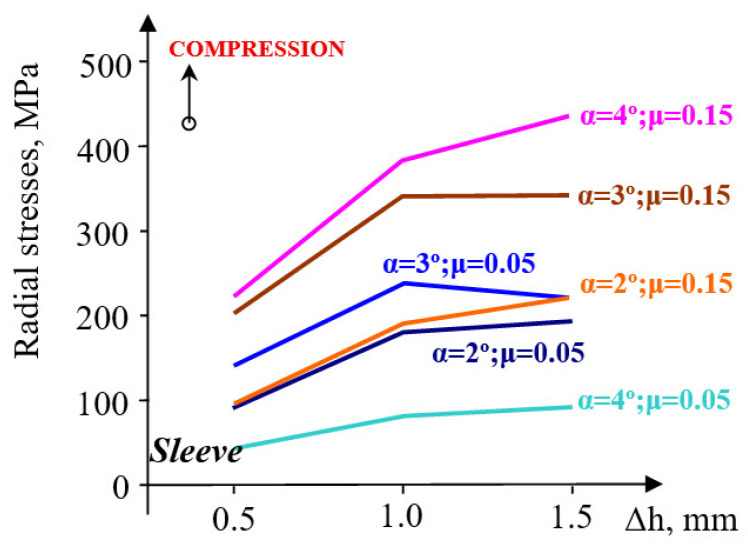
Evolution of radial stresses in the sleeve for varying values of taper angle α and friction coefficient μ.

However, at larger pre-compressions (Δh = 1.0–1.5 mm), radial stresses increase, approximately to the same order of magnitude. At large angles (3–4°), the evolution trend is completely different: stresses increase at small pre-compressions and decrease at higher pre-compressions. A taper angle increase from 3° to 4° produces a rise in radial stresses by 17 MPa, for a friction coefficient μ = 0.05, and only by 4 MPa at μ = 0.15. On the other hand, an increase in the angle from 2° to 3° causes the stresses to increase by about 3.3 times (μ = 0.05) and by 5 times for a friction coefficient of 0.15. The evolution of radial stresses is very sensitive to the increase in the inclination angle from 2° to 3° when the friction coefficient also increases. From this point of view, raising the angle above 3° would not be justified. In the upper-die body, the compressive radial stresses (Figure 14) show a growth trend with increasing pre-compression in the interval Δh = 0.5–1.0 mm and with higher friction coefficients.

**Figure 15:**
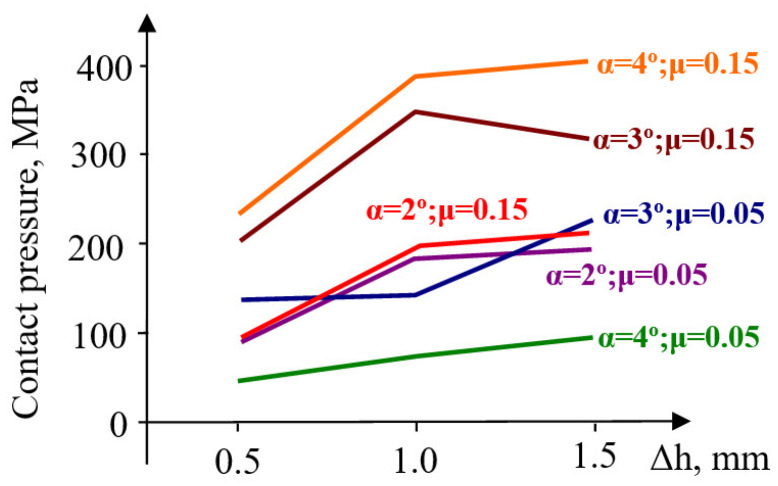
Evolution of sleeve–upper-die body contact pressure for different values of the taper angle α and friction coefficient μ.

At larger pre-compressions (Δh = 1.0–1.5 mm), a contradictory trend of evolution appears: a slight increase at angles of 4°, and a relatively constant or slightly decreasing trend at smaller angles. The contact pressure has an evolution very similar to that of radial stresses (Figure 15), almost across the entire range of pre-compression values used in this evaluation.

This phenomenon may be due to the fact that, at the taper angle of 4°, the elastic sleeve is pushed out of the container in the opposite direction to the pressing force (see Appendix B, Figure A4d). Considering this aspect, it was concluded that the taper angle of 4° is too high and, from a technological point of view, is not of interest.

To avoid the risk of the sleeve jamming inside the upper-die body, for the experimental studies and subsequent finite element analyses, dies were chosen with a sleeve (and upper die body) taper angle of 3°.

### 2.3. Experimental Investigations on Deformation in Elastic Dies

#### 2.3.1. Materials, Equipment, and Experimental Methods

**Figure 16:**
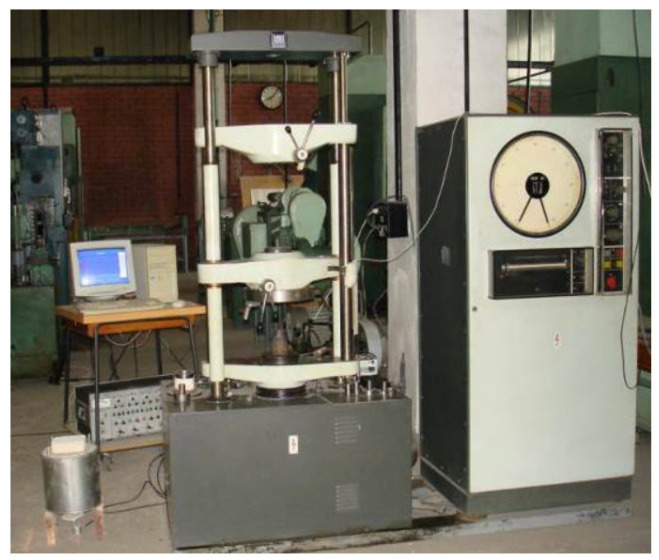
Heckert hydraulic testing machine in the IPM laboratory, Cluj.

**Figure 17:**
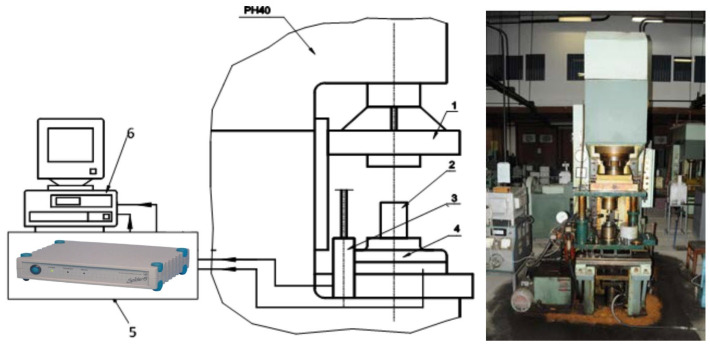
Schematic of the experimental stand for powder compaction. 1—mobile crosshead, 2—die assembly, 3—inductive stroke transducer 100 mm, 4—strain gauge force transducer, 5—SPIDER strain gauge bridge, 6—computer with PC26AT data acquisition card.

The experimental program was conducted in the Laboratory of Materials Processing Engineering (Technical University of Cluj). Trials were performed on a Heckert hydraulic testing machine, produced in Lepzig, Germany by VEB Werkstoffprüfmachinen (up to 200 kN) for preliminary force–stroke measurements (Figure 16) and on the PH-40 compaction stand, Schmidt hydraulic press, produced in St. Georgen, Germany by SCHMIDT Galvanotechnik and STS Schreibgerätetechnik Schwarzwald, for high-load tests up to 325 kN, with synchronized acquisition of force and stroke (Figure 17).

This laboratory is equipped with a stand for powder compaction, consisting of the PH 40 hydraulic press and the necessary instrumentation for recording parameter variations (force, stroke) during the powder compaction process (Figure 17).

The experimental program aimed to use powders with varied characteristics, in order to obtain data providing as broad an understanding as possible of powder compaction. For the compaction tests of cylindrical-shaped parts, the following powders were used:

- Fe normal compressibility (NC)
- Fe super-compressibility (SC)
- Fe–Cu–graphite mixture
- Stainless steel 316L

All powders used in this study (Fe-NC, Fe-SC, Fe–Cu–graphite, and 316L stainless steel) were available in the Laboratory of Materials Processing Engineering at the Technical University of Cluj. The chemical composition (excluding Fe) and granulometric analysis of these powders are presented in Table 1 and Table 2.

The exact production source is not specified. The powders used were not mixed with stearates or other lubricants. The Fe–Cu–graphite mixture was tested both with zinc stearate additive and without, to observe differences in compaction behavior between the two cases. The stainless steel 316L powder (European grade X2CrNiMo17-12-2) was gas-atomized, exhibiting spherical morphology with an average particle size of ≈110 µm and a theoretical density of 7.99 g cm^−3^; its granulometric distribution used in this study is given in Table 2. The Fe–Cu–graphite mixture was assessed both without and with zinc stearate to quantify lubrication effects.

**Figure 18:**
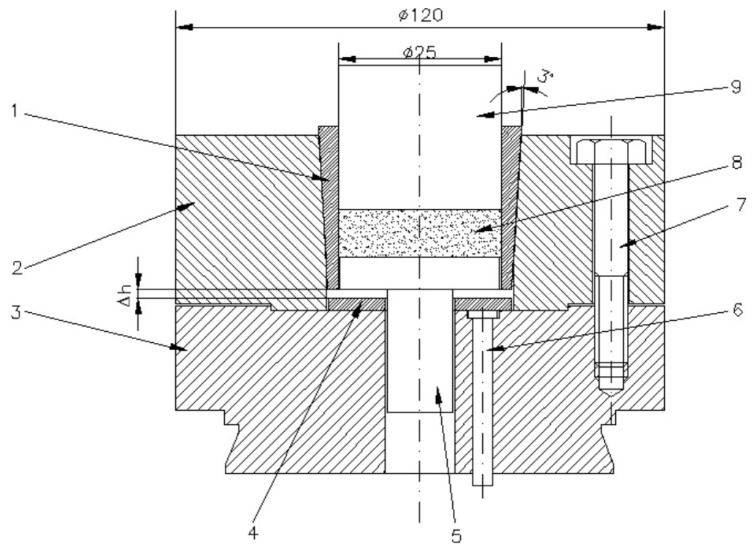
Elastic die assembly used in experiments. 1—elastic die/sleeve; 2—upper die holder; 3—lower die holder; 4—spacer ring; 5—ejector; 6—die ejector rods; 7—assembly screws (3 pcs. at 120°); 8—compact; 9—punch. (the units are millimeters).

Obtaining high-density compacts, especially when the powder is difficult to compress, raises problems due to dimensional relaxation occurring when the compact is extracted from the die. As the compact is gradually removed from the die, the free part relaxes while the part still inside remains compressed, leading to cracks at the boundary between the two zones. To avoid this phenomenon, relaxation must occur simultaneously along the entire height of the compact. For this purpose, the elastic die assembly shown in Figure 18 was designed and developed.

**Figure 19:**
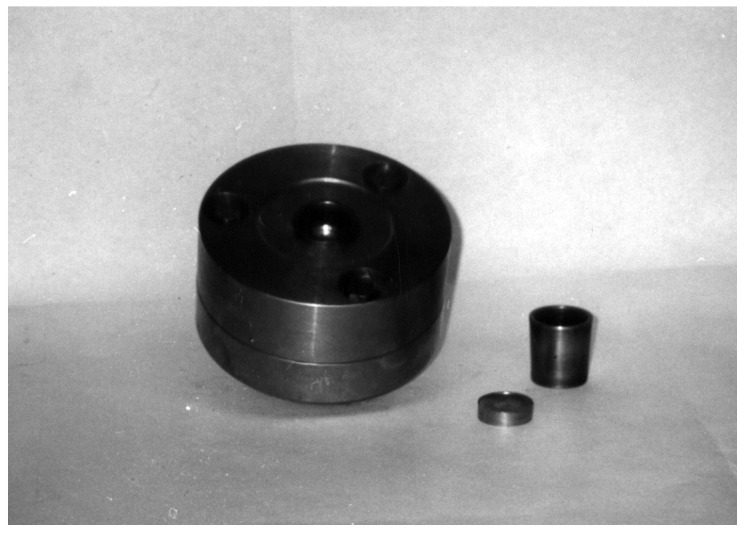
Elastic die assembly and pressed part (photo).

The elastic die (1) has a cylindrical interior and conical exterior; the upper die holder (2) has a conical seat corresponding to the outer surface of the elastic die (1). The elastic die is axially pressed into the upper die holder (2), thus reducing its internal diameter. The reduction value must be approximately equal to the dimensional relaxation of the compact during extraction. This reduction in diameter can be controlled using the spacer ring (4). After the powder compaction process ends, the elastic die is depressed with the help of the ejector rods (6). The elastic recovery of the die and the relaxation of the compact occur simultaneously over their entire height, eliminating the risk of cracks in the part. Moreover, since the die no longer contracts on the compact, extraction requires a reduced force. The image of the obtained part and the die is shown in Figure 19.

The elastic die is axially pressed into the upper die holder, and due to its conicity, it will elastically deform radially, reducing its transverse dimensions by the value given in Equation (2). By using three spacer rings of different heights, three axial pressing depths of the die were obtained: Δh = 0.5 mm, 1 mm and 1.5 mm. This allowed evaluation of the influence of elastic die pre-straining on powder compaction.

Another parameter influencing the density obtained after compaction is the pressing force. To study the influence of pressing force on compact density, powders were pressed under different forces: 250 kN, 275 kN, 300 kN, and 325 kN.

#### 2.3.2. Experimental Results

The density of the compacts was calculated after weighing the samples and determining their volume based on measured geometrical dimensions.

**Figure 20:**
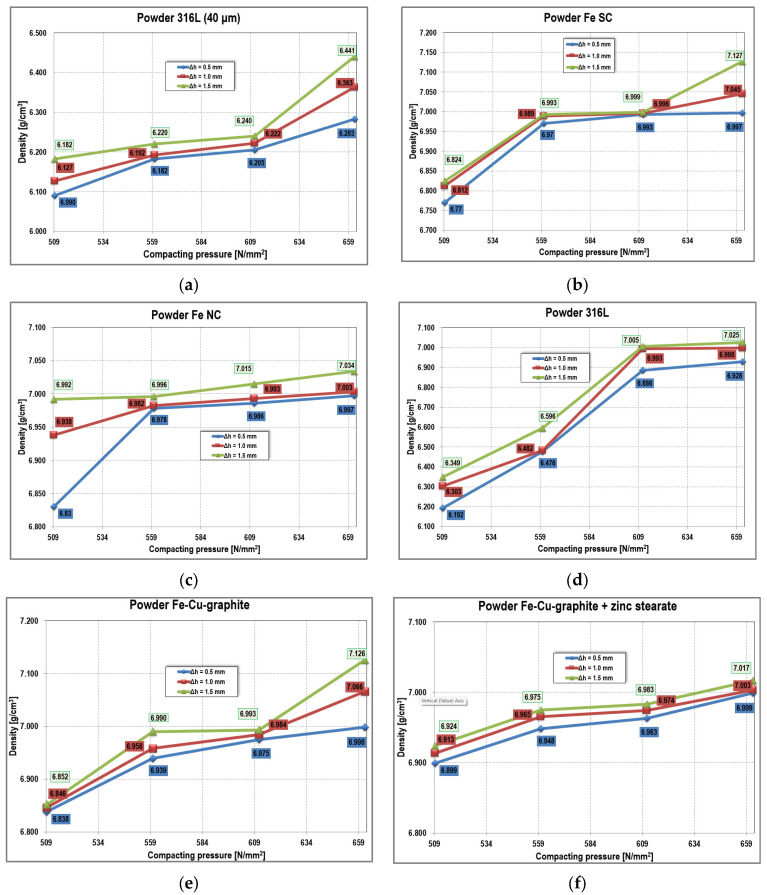
Density evolution at different compacting pressures (**a**) 316L stainless steel powder grain size < 40 µm; (**b**) Iron powder super-compressibility; (**c**) Iron powder normal-compressibility; (**d**) 316L stainless steel powder; (**e**) Iron–Copper–Graphite powder; (**f**) Iron–Copper–Graphite powder + zinc stearate.

The measured geometrical dimensions, mass values, and densities are listed in Appendix C. With these values, graphs were created to show the influence of the parameters considered on the density of the compacted samples. In the graphs presented in Figure 20, the evolution of density depending on the pressing force is studied for different pre-strain values and several types of metallic powders. As a general observation, there is a visible tendency for density to increase with increasing pressing force and pre-strain. A slight linear growth trend in density appears at low pressing force values (250–275 kN):

- 1.5% increase for pre-strain of 0.5 mm
- 0.6% increase for pre-strain of 1.5 mm (Figure 20a).

At higher pressing forces (300–325 kN), density increases more significantly with larger pre-strain values:

- 3.2% increase at pre-strain of 1.5 mm
- 1.2% increase at pre-strain of 0.5 mm.

For Fe SC powder (Figure 20b), density increases more strongly at low pressing forces (250–275 kN), with an increase of 3% for Δh = 0.5 mm and 2.5% for Δh = 1.5 mm. For the largest pre-strain (Δh = 1.5 mm), density also increases by 1.8% at higher force values, i.e., in the range of 300–325 kN. At high force values, density does not change for the smallest pre-strain (Δh = 0.5 mm).

For Fe NC powder (Figure 20c), a significant change appears at a small pre-strain (2.2%) and force in the range 250–275 kN. For all three above analyzed powders, density evolution is approximately linear in the range 275–300 kN, but the increase is insignificant for all pre-strain values.

A different trend is observed for 316L stainless steel powder (Figure 20d), where density increases by 11.2% at Δh = 0.5 mm and 10.3% at Δh = 1.5 mm, for pressing forces in the range 250–300 kN. At higher pressing forces, the increase in density is insignificant regardless of the applied pre-strain.

For Fe–Cu–graphite powder (Figure 20e), density increases by 2.9% (Δh = 0.5 mm), 3.2% (Δh = 1 mm), and 4% (Δh = 1.5 mm).

For the Fe–Cu–graphite + zinc stearate mixture (Figure 20f), the density increase is smaller: 1.4% (Δh = 0.5 mm), 1.9% (Δh = 1 mm), and 1.3% (Δh = 1.5 mm) for pressing forces in the range 250–325 kN. The presence of zinc stearate does not favor compaction, as seen in the comparative analysis of the two graphs (Figure 20e,f).

The relative density shows a slight linear growth trend, more evident for Fe–Cu–graphite powder and 316L stainless steel. Although the pretension value of the sleeve (Δh = 0.5–1.5 mm) does not highlight a significant increase in relative density for the same pressing force value, regardless of the type of metallic powder used, it was nevertheless observed that the compacts could be extracted easily from the die, without difficulties. This demonstrates that the elastic relaxation of metallic powder compacts is smaller than the elastic recovery of the sleeve, fulfilling the functional role for which it was designed.

For the purpose of identifying the material behavior equation, classical (uniaxial) compaction tests were performed using the Heckert hydraulic press (Figure 16). By this method, two types of powders were compacted: Fe and 316L stainless steel (see the results in Table 3).

**Figure 21:**
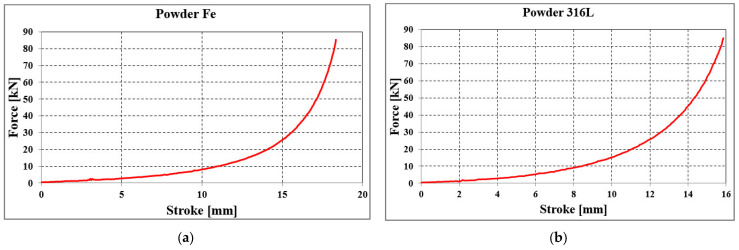
Force–stroke graph for the classical compaction of (**a**) Fe powder and (**b**) 316L stainless steel powder.

Using the installation presented in Figure 16, force–stroke graphs were obtained for each sample, presented in Figure 21.

A linear increase in force is observed, reaching 10 kN at a punch stroke of 12 mm. After this point, the increase becomes exponential, reaching values of 60–80 kN at a stroke of 17–18 mm (Figure 21a).

For stainless steel powder, the linear evolution of force is visible up to a punch stroke of 10 mm (force reaches 11 kN). A further increase in stroke from 10 to 15 mm results in a slower increase in compaction force (60 kN).

### 2.4. Application of the Finite Element Analysis Code—MARC Mentat in Metal Powder Compaction

This subsection reports in detail the finite element model used in the study, including the mesh design and convergence checks, the boundary conditions and frictional contact definitions, as well as the calibrated material parameters for Fe and 316L stainless steel powders (Table 4 and Table 5).

#### 2.4.1. Presentation of Constitutive Equations Used in Numerical Calculation

Powder compaction, in this work, was carried out in a closed die, where deformation of the preform is possible due to the change in volume. The non-uniformity of deformation distribution is caused by inter-particle friction, friction between die and powder, and at the punch–powder contact, which leads to distortions of flow lines.

The simulation was performed using the MSC.Marc Mentat 2005 numerical simulation software. The material behavior model incorporated in MSC.Marc Mentat is of the viscoplastic type. The characteristic equation is [40]:

(1) $$F=\frac{1}{\gamma }\cdot {\left(\frac{3}{2}\cdot \sigma^{d}\cdot \sigma^{d}+\frac{p^{2}}{\beta^{2}}\right)}^{1/2}-\sigma_{y}$$

where

- σ_y_—uniaxial yield stress;
- σ^d^—stress tensor deviator;
- p—hydrostatic pressure;
- γ and β—material parameters.

σ_y_ is a function of temperature and relative density; γ and β are functions only of the relative density ρ.

The material parameter γ is calculated with relation (2):

(2) $$\gamma ={\left(b_{1}+b_{2}\cdot \rho^{b_{3}}\right)}^{b_{4}}$$

In this formulation, γ is the rate parameter appearing in Equation (2), calibrated from force–stroke data in Section 2.4.2. As the powder becomes denser, ρ approaches the value of 1, and the model reverts to the classical von Mises model. It should be noted that elastic properties are also functions of relative density. When the material becomes fully dense, Poisson’s ratio approaches 0.5.

Since most processes involving powdered materials are driven by both pressure and thermal effects, it is necessary to perform a complex analysis.

#### 2.4.2. Identification of Material Behavior Parameters

**Figure 22:**
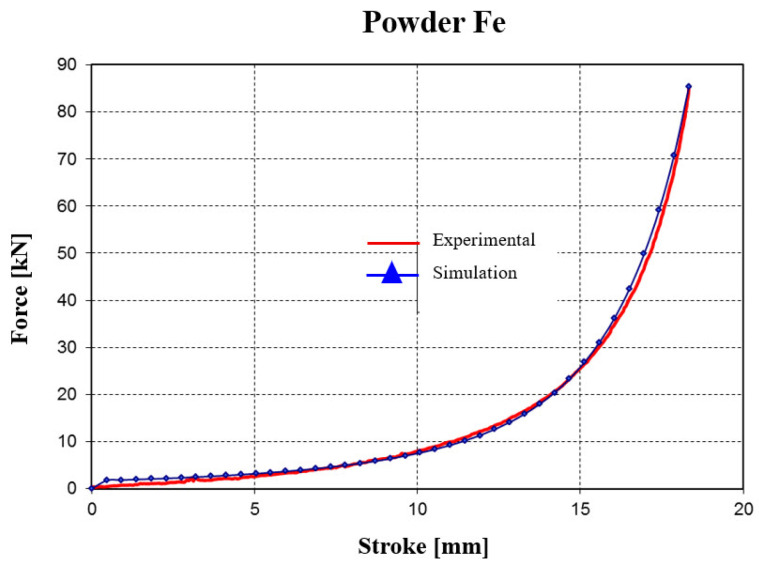
Evolution of the force–stroke curve (for iron powder) obtained from experimental tests and simulations.

By applying the inverse method, the identification of material behavior parameters was performed through interpolation of the curve determined experimentally (see Figure 21a) with the material data available in the MSC.Marc Mentat database (Figure 22). After running several classical (uniaxial) compaction simulations, the identified parameters are:

As a result of identifying the material behavior parameters, an average error of about 15% was obtained between the curve recorded experimentally and the curve resulting from the numerical analysis.

In the following numerical analyses of Fe powder compaction in the elastic die, the material parameters presented in Table 4 will be used.

**Figure 23:**
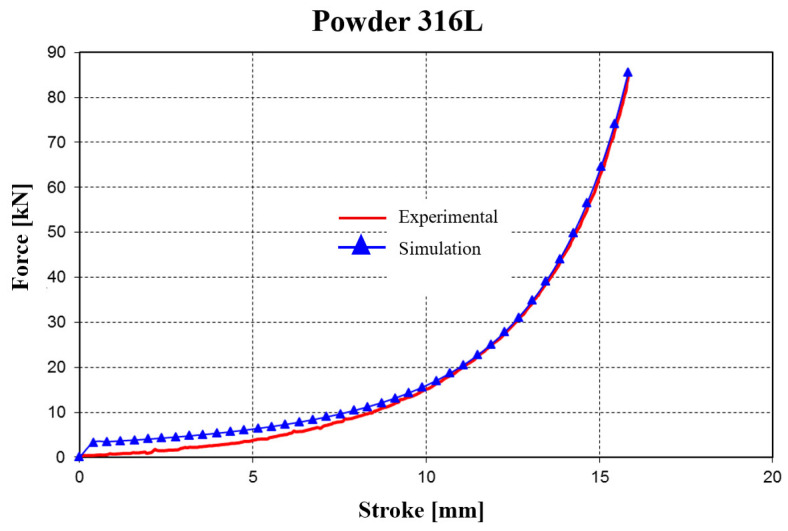
Evolution of the force–stroke curve (for 316L stainless steel powder) obtained from experimental tests and simulations.

Similarly, the procedure was applied for 316L stainless steel powder. The values obtained for the material parameters are presented in Table 5. In this case, the average error was about 20% between the experimentally recorded curve and the curve obtained from the numerical analysis (Figure 23).

#### 2.4.3. Introduction of Boundary Conditions Required for Numerical Analysis

Creation of the geometric model: Since the analysis model is axisymmetric, the finite element modeling of the structure was carried out starting from the closed half-contour of the planar domain. Obtaining this contour involved generating a boundary, composed of lines, that delimits the analysis domain. A Cartesian coordinate system was used to facilitate the generation of model entities.

The boundary of the geometric domain of the model in the working plane is defined by a rectangle. The length of the short sides of the rectangle is given by the radius of the green compact, while the length of the long sides of the rectangle corresponds to its height. The lower boundary of the rectangle represents the axis of symmetry of the studied model.

Discretization (generation of nodes and finite elements) is imposed by defining linear boundaries that delimit the planar domain of the analyzed model through closed contours. In the analyzed models, meshes were generated using ideal square-shaped finite elements (from a geometric point of view) with a dimension of 0.4 mm.

**Figure 24:**
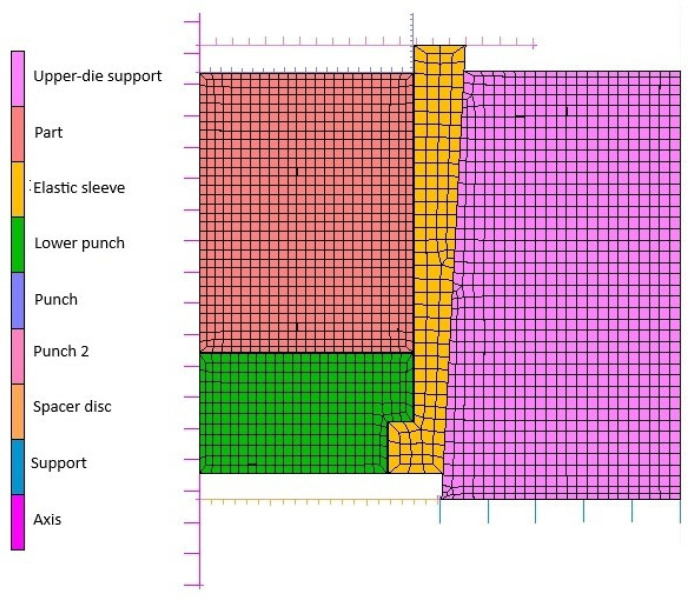
Finite element mesh of the axisymmetric elastic die–powder model.

The resulting finite element mesh and node grid, obtained after defining the discretization characteristics and applying the boundary conditions, are shown in Figure 24.

As can be seen in Figure 24, to obtain results as close as possible to the real powder compaction process, the part, elastic sleeve, lower punch, and upper-die support were considered deformable. The remaining elements were considered rigid bodies.

The initial relative density is considered by the calculation code as a user-defined variable. Therefore, the numerical calculation for 316L stainless steel was performed for an initial relative density of 0.37.

For defining the friction conditions, a Coulomb-type friction model was used in the numerical analysis, characterized by Equation (3) [36]:

(3) $$\sigma_{t}=-\mu \cdot \sigma_{n}\cdot t$$

where

σ_t_—tangential friction stress
σ_n_—normal stress
μ—friction coefficient
t—tangential vector in the direction of relative velocity

Since experimentally it was not possible to measure the friction parameters between the metallic powder and the die walls, in the numerical calculation, the following approximations were made:

- coefficient of friction between metallic powder particles and elastic sleeve: 0.15
- coefficient of friction between the other deformable components: 0.1

Compaction of cylindrical preforms from 316L stainless steel powder was performed on a hydraulic press. The upper punch speed in the vertical plane was set to 1 mm/s, like the value used in the experimental tests.

As in the experimental trials, three spacer rings of different heights were used, resulting in three axial pressing depths of the die: Δh = 0.5 mm, 1.0 mm, and 1.5 mm. This allowed evaluation of the influence of elastic die pre-straining on powder compactness.

#### 2.4.4. Simulation of Cylindrical Preform Compaction

**Figure 25:**
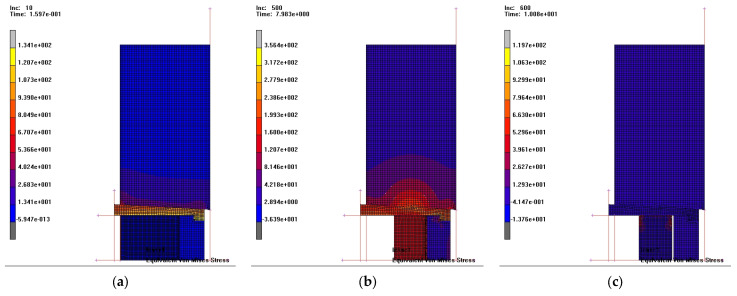
Distribution of equivalent von Mises stresses: (**a**) initial stage; (**b**) end of compaction and (**c**) extraction phase.

In Figure 25, the distribution of equivalent von Mises stresses is shown in the deformed part and in the die, for compaction with pressing depth Δh = 0.5 mm, at three distinct stages of the compaction process:

- at the beginning of compaction (increment 10),
- at the end of compaction—when the elastic sleeve is in contact with the spacer ring (increment 500),
- and in the final stage of the extraction phase—after removal of the upper punch, when the sleeve relaxes elastically (increment 600).

At the initial stage, a uniform stress state was initiated throughout the part with values ranging between 40.2 and 67.0 MPa, and at the surface in contact with the punch, die, and sleeve, initial values of 13.4–26.8 MPa.

As the process developed, the von Mises stress values increased and reached:

- 199.3–277.9 MPa in the part,
- 42–160 MPa in the lower punch.

The stress distribution in the lower punch is very non-uniform along its height, with high values (160 MPa) at the surface in contact with the part and much lower values (42 MPa) at a distance of 5–8 mm along its height.

In the elastic sleeve (Figure 25b), the stress values are relatively uniform (81.4–120.7 MPa). In the extraction phase (Figure 25c), the equivalent stress values approach zero throughout the entire die assembly, indicating the elastic relaxation that occurs at this stage.

**Figure 26:**
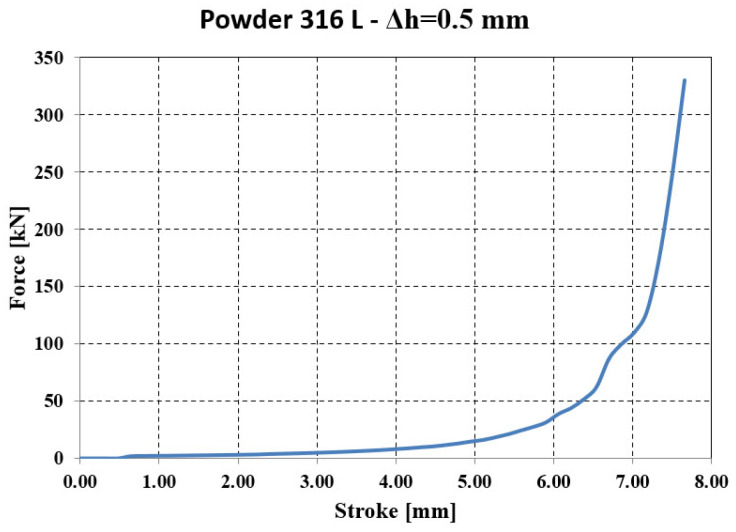
Force—stroke evolution resulting from numerical analysis.

The compaction force (Figure 26) is relatively stable at the beginning of the process, with an increase of only 30–40 kN for a stroke of 4.0–6.0 mm, but it rises sharply in the final phase (45–300 kN) even though the punch stroke is only 1.0–1.5 mm.

**Figure 27:**
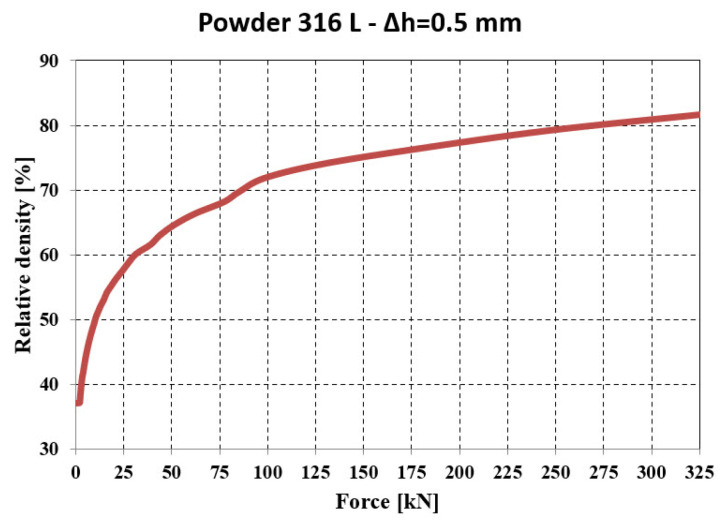
Evolution of relative density—force resulting from numerical analysis.

The relative density (Figure 27) evolves in accordance with the pressing force and reaches a value of 81.87% at the maximum force, which is close to that obtained in the experimental tests—86.71% (see Figure 20d).

Increasing the pressing depth (Δh = 1.0 mm) leads to a modification of the equivalent stresses developed in the die assembly, elastic sleeve, part, and lower punch.

At the beginning of compaction, von Mises stresses in the part range between 56.4 and 70.5 MPa, and between 28.2 and 42.3 MPa at the contact surfaces with the punch and die, showing a relatively uniform distribution. At the end of compaction, the stress values are between 322 and 497 MPa in the part, and between 237 and 322 MPa at the punch–part contact surface. Here, the spread of high values is much reduced and localized near the active tool surfaces. In the extraction phase, the stresses tend to approach zero in the entire analyzed assembly. The remaining elastic stresses are reduced, with values between 12.9 and 26.2 MPa.

The deformation force changes insignificantly up to a stroke of 5.0–6.0 mm, then increases exponentially in the final phase of compaction (300–350 kN), while the punch stroke increases by only 1.0–2.0 mm. The relative density exceeds 70% even at low pressing forces (50–75 kN), but the threshold of 80% relative density is reached only at forces above 225 kN. The maximum value, 83.27%, is achieved at 325 kN. For comparison, in the experimental tests, a relative density close to 87.58% was obtained.

For a compaction process with a pressing depth of Δh = 1.5 mm, the distribution of von Mises equivalent stresses is similar to the previous technological variants, but their intensity differs. At the initial stage of compaction (increment 10), von Mises stresses between 53.3 and 62.2 MPa develop in the processed part. At the end of the compaction phase, stresses in the part range between 370 and 521 MPa, with variable values along the height of the punch. These values decrease from the contact surface with the part (671 MPa) to about 69.4 MPa at 15–20 mm along the punch height.

As expected, in the extraction phase, elastic relaxation of both the die components and the green compact is observed, shown by stress values close to zero.

In this analyzed variant, the force increases significantly, reaching 300–350 kN, even though the punch stroke is only 1.0 mm. This sharp increase is specific to the final compaction phase.

In the force range of 175–325 kN, the relative density increases from 79% to 85.8%, which suggests that doubling the pressing force produces only a relatively small change in density. It is worth analyzing whether such an intense mechanical load brings sufficient benefits in terms of density increase.

The relative density obtained from numerical analysis was 85.80%, while from the experimental tests (see Section 2.3.1), a relative density of 88.86% was obtained (Table 6).

The average error in terms of relative density in these three cases is 4.6% (between the values obtained experimentally and those resulting from numerical analysis).

The comparative evolution of the compaction force for different pressing stroke values suggests the same overall trend, with the remark that by increasing the pressing depth, the steep slope section of the curve shifts slightly, moving closer in value to the pressing stroke.

In all three cases, the abrupt increase in force from about 150 to 350 kN occurs with an increase in pressing depth of less than 0.5 mm. This evolution underlies the mechanism of easy extraction of green compacts from the elastic die.

#### 2.4.5. Comparison of the Theoretical Model with the Experimental Results

In the experimental determination, the shape of the pressing force evolution curve differs noticeably from that obtained through the simulation procedure, but the two curves converge in the final pressing phase.

A significant difference appears for strokes between 4 and 7 mm, where the curve obtained in the simulation procedure is much more flattened.

This difference, which arises in the intermediate stage of the compaction process, is explained by the limitations of the viscoplastic model used in the simulation procedure, which cannot account for the increasing friction within the mass of the forming compact.

On the other hand, the equipment used for the experimental trials has a specific mechanical response dictated by the size of the load and the testing conditions, which cannot be quantified with sufficient accuracy to be fully incorporated into the simulation procedure.

The FEM predictions were validated against the experimental compaction results presented in Section 2.3. Quantitative comparisons were made for force–stroke curves and final relative density values across all powders and spacer settings (Δh = 0.5–1.5 mm). The average absolute error in predicted final relative density was approximately 4.6%. In terms of compaction force, the FEM model reproduced the general trends but underestimated the force in the intermediate stroke region by about 15–20%. This discrepancy is consistent with limitations of continuum formulations in representing particle rearrangement and evolving wall friction, as reported in the literature. Overall, the validation confirms that the FEM model provides reliable predictions of densification behavior under elastic die compaction.

## 3. Results

### 3.1. Numerical Analysis of the Elastic Die Assembly

Finite element simulations in SolidWorks Simulation were performed to evaluate the stress distribution and contact mechanics between the elastic sleeve and the rigid holder. The results indicate a strong dependence on the conical taper angle (α), the axial pretension (Δh), and the friction coefficient (μ).

- Contact pressure: At Δh = 1.0 mm and μ = 0.10, the average contact pressure increased from ≈52 MPa (α = 1°) to a maximum of ≈200 MPa (α = 3°), followed by a slight reduction at α = 4°. Thus, a taper of 3° yields the most favorable stress transfer between the sleeve and holder, defined here by achieving both the highest average contact pressure (≈200 MPa) and a relatively uniform pressure distribution along the conical interface, without excessive stress concentration near the sleeve edges.
- Circumferential stresses: In the elastic sleeve, hoop stresses increased nearly linearly with Δh, reaching values above 900 MPa at Δh = 1.5 mm for α = 3°. The corresponding holder stresses were lower by ≈40–50%, confirming effective load sharing.
- Frictional influence: Increasing μ from 0.05 to 0.15 shifted the stress distribution toward the sleeve and reduced the effective radial compression on the powder, highlighting the importance of lubrication or surface finishing in the die–sleeve interface.

These results demonstrate that the elastic conical contact provides controlled radial confinement, with α ≈ 3° and Δh = 1.0–1.5 mm representing the optimal design window.

### 3.2. Constitutive Model Identification and Validation

The viscoplastic constitutive model for powder compaction was calibrated using uniaxial force–stroke experiments on stainless steel 316L powder. The model parameters (b_1_–b_4_, β) were obtained by least-squares fitting of the measured curves. The full calibrated set for each powder (including γ) is reported in Table 4 and Table 5, together with elastic constants and yield stress.

- Force–stroke curves: Simulations reproduced the experimental compaction curves with an average force prediction error of 15–20%, with the largest deviations occurring in the mid-stroke region.
- Density evolution: The predicted relative density–force relationship showed a mean error of ≈4.6% across the compaction range, confirming that the model is sufficiently accurate for subsequent parametric studies.
- Mesh sensitivity: Refinement studies confirmed that the predicted density distribution stabilized with <5% variation once the element size in the powder domain was reduced below 0.5 mm.

### 3.3. Experimental Compaction of Metallic Powders

- Experimental validation was conducted on four powders: Fe normal compressibility (NC), Fe super-compressibility (SC), Fe–Cu–graphite mixture, and 316L stainless steel, each tested with and without zinc stearate lubrication. Cylindrical compacts were produced under applied forces up to 325 kN, using spacers to impose Δh values of 0.5, 1.0, and 1.5 mm.
- Relative density trends: For 316L, density increased from an initial ρ_0_ ≈ 0.37 to a maximum of ρ ≈ 0.889 at Δh = 1.5 mm and 325 kN. For Fe–Cu–C, the final density reached ρ ≈ 0.865, while Fe-NC and Fe-SC exhibited lower densification levels under identical conditions.
- Effect of pretension (Δh): Increasing Δh consistently enhanced density by ~3–5% at the same pressing force, confirming that higher sleeve pre-compression improves radial support.
- Effect of lubrication: The addition of zinc stearate lowered the compaction force by ≈10–15% for a given density but did not significantly change the maximum achievable density.

### 3.4. Correlation of Numerical and Experimental Results

Comparisons between finite element predictions and experimental measurements highlight the following:

- Density agreement: Simulated final densities matched experimental values within ±5% for both 316L and Fe–Cu–C compacts. At 325 kN and Δh = 1.5 mm, experiments yielded ρ ≈ 0.889 for 316L and ρ ≈ 0.865 for Fe–Cu–C, while simulations predicted ρ ≈ 0.895 and ρ ≈ 0.870, respectively. This confirms that the calibrated material parameters reproduce densification trends with good accuracy.
- Force–stroke correlation: The FEM-predicted compaction curves followed the experimental load–displacement profiles closely. For Fe-based powders, the average deviation was ≤15%, and for 316L, ≈20%. Differences were most pronounced in the mid-stroke region, where particle rearrangement and local friction effects, not explicitly captured by the constitutive model, contributed to higher experimental forces (Figure 22 and Figure 23).
- Stress evolution: The predicted circumferential stress distribution in the sleeve aligned qualitatively with strain-gauge measurements on the external surface. Both experiment and simulation identified peak hoop stresses near the mid-height of the sleeve. While quantitative strain validation requires additional tests, the current results support the FEM stress field predictions.
- Design optimization: The combined numerical–experimental evidence points to an optimal die geometry with α = 3° and Δh = 1.0–1.5 mm, balancing high density, controlled stresses, and favorable relaxation behavior that facilitates extraction.

## 4. Discussion

### 4.1. Influence of Taper Angle on Stress Transfer

The finite element analysis revealed that the contact pressure between the elastic sleeve and the rigid holder increases sharply with α, reaching a maximum near 3°. This optimum can be explained by the balance between radial confinement and axial slip.

- At small angles (α = 1–2°), the conical interface provides insufficient radial expansion, resulting in limited sleeve pre-stress and reduced powder confinement.
- At larger angles (α = 4°), the contact area shortens, and local sliding dominates, reducing uniformity of radial pressure.

The observed optimum near 3° can be explained by the balance between radial confinement and axial slip, a mechanism also reported in conical shrink-fit die studies [24,27], which reported comparable taper values as critical for stable stress transfer.

### 4.2. Role of Axial Pretension (Δh)

Increasing axial interference (Δh) systematically increased both sleeve stresses and powder density, confirming that controlled pre-compression enhances radial support. However, the efficiency of densification decreased beyond Δh = 1.0 mm, as density gains diminished despite higher sleeve stresses (>900 MPa). The underlying mechanism is that once the sleeve is already highly prestressed, additional axial interference is absorbed mainly as elastic strain in the sleeve rather than being effectively transmitted as radial pressure to the powder. At the same time, the compact is approaching its packing limit, so further densification requires extensive particle rearrangement and plastic deformation, which are less sensitive to small increments of radial confinement. This combination explains the diminishing returns observed, while also pushing the sleeve closer to its elastic durability limit. Thus, Δh = 1.0–1.5 mm appears optimal, balancing stress, density, and sleeve durability.

### 4.3. Frictional Effects and Implications for Ejection

Friction at the sleeve–holder and die–powder interfaces critically affects both stress distribution and extraction.

- Higher μ values transferred more stress to the sleeve but reduced the effective radial compression on the powder, lowering densification efficiency.
- From an operational perspective, upon unloading, the elastic sleeve relaxes and the wall pressure drops, which reduces the ejection effort. This behavior is supported by our Marc Mentat analysis: Figure 25c shows the von Mises stress distribution in the sleeve during the extraction phase, confirming a relaxation of stresses at the powder–die interface. Experimentally, the green parts were removed without sticking, although ejection forces were not instrumented. In line with prior balanced-die literature [25], we conclude that the elastic die design lowers ejection effort compared with rigid dies. Although the present study inferred easy ejection indirectly, future work should quantify the ejection force experimentally, since prior studies reported force reductions of up to 70% compared with rigid dies.

### 4.4. Material and Lubrication Effects

The experimental results demonstrate that powder composition strongly influences densification behavior.

- 316L stainless steel achieved the highest final density (ρ ≈ 0.889), consistent with its finer particle morphology and greater compressibility.
- Fe–Cu–C powders compacted to slightly lower final densities, while Fe-NC and Fe-SC exhibited more limited densification, reflecting differences in flowability and particle bonding.

The addition of zinc stearate lowered compaction forces by ~10–15% but had minimal impact on maximum density. This result aligns with lubricant-assisted die compaction studies [28], which highlighted improved processability but negligible changes in densification limit.

### 4.5. Correlation Between Simulation and Experiment

The numerical predictions reproduced experimental density–force trends within ±5%, demonstrating that the identified constitutive model captures the main powder consolidation mechanisms. However, force–stroke errors of 15–20% in the mid-stroke region suggest that additional phenomena, such as particle rearrangement or local die wall compliance, are not fully represented in the current constitutive law.

Nevertheless, the validated model is sufficiently accurate for design optimization of elastic dies, particularly when evaluating the relative effects of α, Δh, and μ.

### 4.6. Practical Implications

The combination of parametric finite element analysis and experimental compaction provides a robust framework for designing elastic dies that minimize ejection forces and improve density uniformity.

Key implications are:

- Taper angle near 3° yields the most efficient stress transfer.
- Pretension Δh = 1.0–1.5 mm enhances densification without overstressing the sleeve. However, excessive interference (beyond 1.5 mm) provides diminishing density gains while driving sleeve stresses above 900 MPa, which could compromise long-term durability. This conclusion is supported by parametric FEM analyses and by compaction tests performed at Δh = 0.5, 1.0, and 1.5 mm on both 316L and Fe–Cu–C powders under forces up to 325 kN.
- Lubrication reduces pressing force but does not significantly affect final density.

## 5. Conclusions

This study demonstrated that the use of elastic-sleeve dies in metal powder compaction provides significant advantages over rigid dies. Numerical simulations showed that a taper angle of 3° and an axial pretension of Δh = 1.0–1.5 mm represent an optimal design window, yielding maximum contact pressure (~200 MPa) and controlled sleeve stresses (<900 MPa) without overloading the holder. Experimental compaction of Fe-based and stainless steel powders confirmed that relative densities up to 0.889 (316L at 325 kN) can be achieved, with pretension increasing densification efficiency by ~3–5%. The addition of zinc stearate reduced compaction forces by ~10–15% but did not alter the final density. Finite element predictions reproduced experimental density–force trends within ±5%, supporting the validity of the proposed constitutive model for design purposes. Although direct ejection-force measurements remain to be carried out, the observed stress relaxation of the sleeve strongly suggests a reduction in extraction effort compared with conventional rigid dies. Overall, the findings establish elastic dies as a promising route for enhanced densification, reduced wear, and easier ejection in powder metallurgy applications.

## Figures and Tables

**Table 1:** Chemical composition.

Material	C [%]	Ni [%]	Cu [%]	Mo [%]	Cr [%]
Fe NC	0.01	-	-	-	-
Fe SC	0.01	-	-	-	-
Fe-Cu-graphite	0.30	-	3	-	-
316L	0.03	10–14	-	2–3	16–18

**Table 2:** Granulometric analysis (particle size in μm).

Material	>160 [%]	160–100 [%]	100–63 [%]	63–40 [%]	<40 [%]
Fe NC	5	38	32	15	10
Fe-Cu-graphite	5	35	40	13	7
316L	7	15	18	28	32

**Table 3:** The measured values of the geometrical dimensions, values of mass and density.

Powder Type	Mass [g]	Diameter [mm]	Height [mm]	Density [g/cm^3^]	Relative Density
316L1 ^1^	8.01	11.19	11.87	6.861706	0.858787
316L2	8.057	11.19	11.98	6.838595	0.855894
Fe1 ^2^	8.015	11.18	11.19	7.296260	0.934220
Fe2	7.991	11.18	11.13	7.313628	0.936444

^1^ Entries labeled “316L1” and “316L2” denote replicate specimens of the same powder batch. ^2^ Entries labeled “Fe1” and “Fe2” denote replicate specimens of the same powder batch.

**Table 4:** Identified material parameters for Fe powder.

Young’s Modulus [MPa]	Poisson’s Ratio	Yield Stress [MPa]	γ ^1^, Coefficients				β
			b_1_	b_2_	b_3_	b_4_	
210,000	0.297	260	0.001	2.85	5.2	0.685	1

^1^ γ is the rate parameter in Equation (2); b_1_–b_4_ and β are material constants of the viscoplastic law (units per Equations (1) and (2)). Average fitting error refers to the force–stroke comparison shown in Figure 22.

**Table 5:** Identified material parameters for 316L powder.

Young’s Modulus [MPa]	Poisson’s Ratio	Yield Stress [MPa]	γ ^1^, Coefficients				β
			b_1_	b_2_	b_3_	b_4_	
196,000	0.297	337	0.001	3.2	5.2	0.65	1

^1^ γ is the rate parameter in Equation (2); b_1_–b_4_ and β are material constants of the viscoplastic law (units per Equations (1) and (2)). Average fitting error refers to the force–stroke comparison shown in Figure 23.

**Table 6:** Maximum relative density values: experimental vs. numerical.

Material 316L	Relative Density [%]		
	Δh = 0.5 mm	Δh = 1 mm	Δh = 1.5 mm
Experimental	86.71	87.58	88.86
Numerical analysis	81.87	83.29	85.80

**Figure A1:**
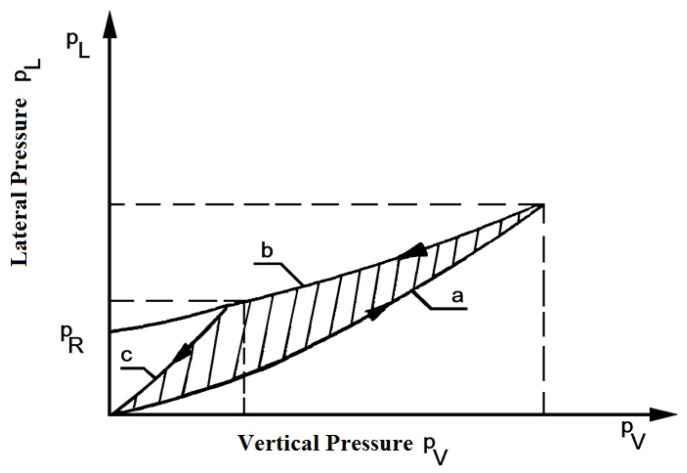
The Pressure distribution at compaction: a—pressing; b—relaxing in rigid die; and c—relaxing at equal pressures.

**Figure A2:**
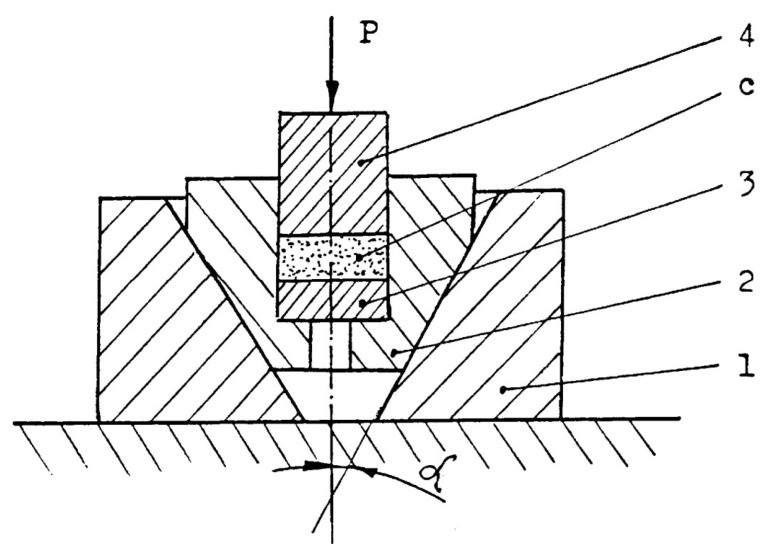
The Compaction Scheme in an elastic die: 1—container; 2—sleeve; 3—ejector; 4—punch; c—powder compact (P—vertical pressure, α—taper angle).

**Figure A5:**
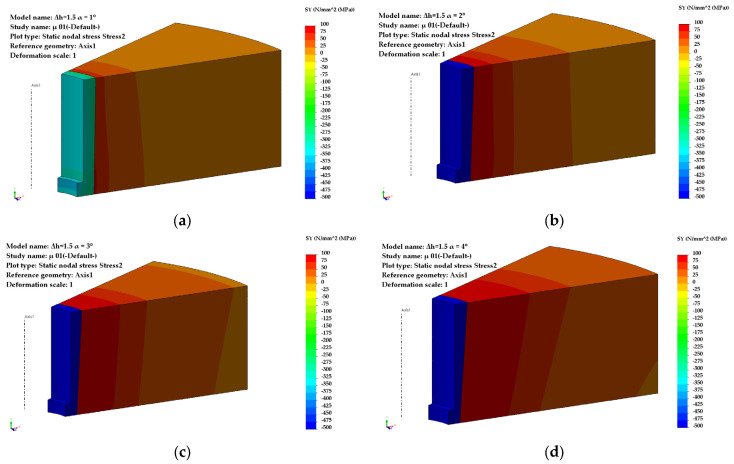
Distribution of circumferential stresses (Δh = 1.5 mm): (**a**) α = 1°, (**b**) α = 2°, (**c**) α = 3°, (**d**) α = 4°.

**Figure A6:**
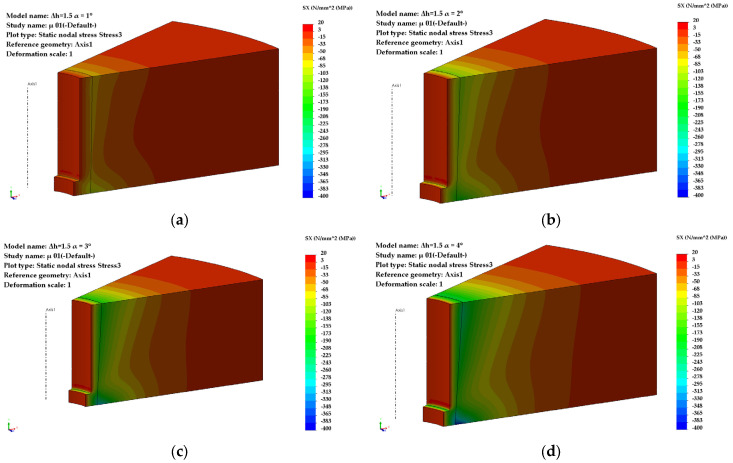
Distribution of radial stresses (Δh = 1.5 mm): (**a**) α = 1°, (**b**) α = 2°, (**c**) α = 3°, (**d**) α = 4°.

## Data Availability

The original contributions presented in this study are included in the article. Further inquiries can be directed to the corresponding author.

## Appendix A

This appendix provides the full derivations and historical schematics originally included in Section 2.1, retained here for completeness.

The elastic die ensures uniform relaxation of residual stresses in all directions after powder pressing or preform die molding, preventing cracks and enabling extraction with significantly reduced friction. Slip cracks occur not only in complex-shaped parts but also in simple cylindrical and ring-shaped parts with flat frontal faces. Exfoliation and cracks are especially common in hard materials with low plasticity, such as carbides, borides, and ferrites. The possibility of these defects increases with density beyond a certain minimum value determined by the compaction pressure. There is a critical compaction pressure at which cracks inevitably form when using rigid dies. The value of this pressure depends on factors such as the material, powder quality, shape, and size of the pressed parts. The risk of exfoliation and cracking can be reduced either by increasing the tensile strength of the workpiece material—by selecting high-quality powder and adding an optimal binder—or by reducing the compaction pressure. However, these measures do not always guarantee the high densities needed for superior mechanical properties in sintered parts. The most favorable conditions for crack formation arise immediately after the pressing process is completed, when the pressing force is released. As the vertical pressing pressure decreases, lateral pressure also relaxes, but at a slower rate. During this stress relaxation in the compact, there is a moment when the decrease in vertical pressure equals that of radial pressure. At this point, the vertical pressure continues to drop to zero, while the lateral pressure reaches its residual radial pressure value, as in Figure A1 [11].

The value of the lateral residual pressure is determined by the elastic deformation of the die and compact. The elastic deformation of the die must occur in such a way that the ratio between the lateral pressure P_L_ and the vertical pressure P_V_ is maintained between the limits:

(A1) $$\xi <\frac{P_{L}}{P_{V}}<1$$

where ξ is the factor of lateral pressure.

The lower limit of this ratio is the degree of compaction, dependent on density (for density, ρ = 1 and ξ = 1). If this ratio decreases by decreasing the lateral pressure, faster than the vertical one, cracks will develop in the pressed part, without the lateral support, initiated by vertical pressure. The upper limit is limited by the condition that during the discharge, the pressure should be smaller than the vertical pressure. Based on the two limits, there is a range of permissible values for the ratio between lateral and vertical pressure, as can be seen in Figure A1 [11] (hatched area), demonstrating the direct dependence between lateral and vertical pressure during the relaxation period. It is obvious that a sufficient condition regarding the elastic deformation of the flexible die is that it is made in such a way as to produce a pressure when relaxed larger than the vertical one. Reference [11] presents an apparatus for producing compacted preforms by compression of metal powder, as seen in Figure A2.

A die is formed of three units and has a slotted elastic shell inside the die. The external surface of the die is conical and is enclosed in a corresponding conical hole of a push ring. Axial movement of the push ring clamps the units of the die towards each other and compresses the slotted shell, closing its slot. Then, metal powder is charged into the shell acting as an inner liner of the die, and the powder is compacted by a single-ended pressing punch. After compaction, the push ring is pressed downward, which opens the die, releasing the compacted preform.

The problem of flexible die displacement during relaxation can be solved either by using a press specially equipped with vertical cylinders (and side cylinders with the possibility of individual programming of their action), or by using elastic dies with the elastic radial deformation determined by the axial pressing movement of the main punch. If the taper angle α is greater than the angle of friction between the die holder and the elastic die, the compaction pressure is transferred through the workpiece to the bottom of the flexible mold, determining its axial pressure and therefore the elastic contraction in the radial direction, of magnitude:

(A2) ∆d=2·∆h·tgα

where ∆d is radial shrinkage per diameter, ∆h is the axial die press and α is the taper angle.

During the relaxation phase, when the upper punch is retracted, the elastic die lifts under the action of lateral pressure by sliding on the inclined faces of the die holder. The optimum value of the taper angle α can be determined from the equilibrium condition (at the limit) of the forces projected on the pressure-relaxation direction, according to the relationship:

(A3) $$tg\left(\alpha -\varphi \right)=\frac{S_{F}}{S_{L}-S_{F}\cdot tg\varphi }$$

where α is the taper angle, φ is the angle of friction, $S_{F}$ is the area of the front surface of the compacted part and $S_{L}$ is the area of the side surface of the workpiece. By fulfilling the condition given by Equation (A3), crack-free parts are obtained due to uniform relaxation of the residual deformations during pressing.

## Appendix B

**Table A1:** Values of circumferential stresses [MPa] for a friction coefficient of 0.05.

Taper Angle	Δh = 0.5 mm		Δh = 1 mm		Δh = 1.5 mm	
	Sleeve	Upper Die Support	Sleeve	Upper Die Support	Sleeve	Upper Die Support
1°	−115.212	25.304	−244.665	57.006	−368.71	84.072
2°	−236.037	48.165	−502.444	105.286	−759.438	153.513
3°	−359.983	67.569	−674.493	138.892	−984.372	174.426
4°	−130.17	26.103	−258.956	48.144	−384.192	68.639

**Table A2:** Values of radial stresses [MPa] for a friction coefficient of 0.05.

Taper Angle	Δh = 0.5 mm		Δh = 1 mm		Δh = 1.5 mm	
	Sleeve	Upper Die Support	Sleeve	Upper Die Support	Sleeve	Upper Die Support
1°	−51.869	11.596	−102.28	4.118	−100.289	5.133
2°	−88.291	19.963	−179.284	10.602	−189.932	15.329
3°	−136.532	22.111	−231.89	35.055	−255.906	11.595
4°	−47.611	7.899	−76.964	18.38	−95.058	5.361

**Table A3:** Values of contact pressure [MPa] for a friction coefficient of 0.05.

Taper Angle	Δh = 0.5 mm	Δh = 1 mm	Δh = 1.5 mm
1°	52.071	102.676	100.293
2°	88.78	181.397	190.0
3°	138.465	139.465	226.034
4°	48.228	75.497	93.149

**Table A4:** Values of circumferential stresses [MPa] for a friction coefficient of 0.1.

Taper Angle	Δh = 0.5 mm		Δh = 1 mm		Δh = 1.5 mm	
	Sleeve	Upper Die Support	Sleeve	Upper Die Support	Sleeve	Upper Die Support
1°	−115.044	26.326	−244.191	59.056	−367.729	89.021
2°	−235.988	50.074	−501.794	111.21	−757.611	163.674
3°	−443.122	87.342	−853.437	192.745	−1250.743	253.406
4°	−576.779	108.207	−1138.79	203.737	−1689.746	290.392

**Table A5:** Values of radial stresses [MPa] for a friction coefficient of 0.1.

Taper Angle	Δh = 0.5 mm		Δh = 1 mm		Δh = 1.5 mm	
	Sleeve	Upper Die Support	Sleeve	Upper Die Support	Sleeve	Upper Die Support
1°	−54.676	12.165	−106.465	3.911	−106.078	4.983
2°	−93.229	20.976	−186.698	10.511	−203.712	14.839
3°	−191.395	30.433	−328.901	51.048	−321.325	12.993
4°	−212.64	39.0	−370.524	55.475	−400.526	20.761

**Table A6:** Values of contact pressure [MPa] for a friction coefficient of 0.1.

Taper Angle	Δh = 0.5 mm	Δh = 1 mm	Δh = 1.5 mm
1°	54.947	106.984	103.913
2°	93.917	189.261	200.129
3°	194.818	333.455	309.841
4°	217.255	378.756	391.85

**Table A7:** Values of circumferential stresses [MPa] for a friction coefficient of 0.15.

Taper Angle	Δh = 0.5 mm		Δh = 1 mm		Δh = 1.5 mm	
	Sleeve	Upper Die Support	Sleeve	Upper Die Support	Sleeve	Upper Die Support
1°	−114.924	27.49	−243.858	60.87	−367.011	94.665
2°	−235.621	52.071	−500.932	111.455	−755.804	172.685
3°	−442.887	90.563	−851.885	202.3	−1247.318	273.03
4°	−576.697	112.561	−1138.07	216.719	−1686.769	312.477

**Table A8:** Values of radial stresses [MPa] for a friction coefficient of 0.15.

Taper Angle	Δh = 0.5 mm		Δh = 1 mm		Δh = 1.5 mm	
	Sleeve	Upper Die Support	Sleeve	Upper Die Support	Sleeve	Upper Die Support
1°	−57.53	12.706	−110.674	4.148	−114.568	4.825
2°	−96.619	22.28	−191.691	10.236	−218.97	14.187
3°	−199.092	31.369	−338.781	53.33	−343.341	12.396
4°	−222.684	41.921	−381.604	57.553	−435.863	20.017

**Table A9:** Values of contact pressure [MPa] for a friction coefficient of 0.15.

Taper Angle	Δh = 0.5 mm	Δh = 1 mm	Δh = 1.5 mm
1°	57.873	111.325	107.608
2°	97.518	194.713	208.347
3°	203.242	344.553	319.891
4°	228.414	391.632	405.584

## Appendix C

Measured values of geometric dimensions as well as mass and density values in the case of cylindrical-shaped parts.

**Table A10:** Δh = 0.5 mm; F = 250 kN.

Powder Type	Diameter [mm]	Height [mm]	Mass [g]	Density [g/cm^3^]
316L (40 μm)	24.94	9.81	29.1873	6.090
Fe SC	24.95	9.10	30.1237	6.770
Fe NC	24.95	10.56	35.2633	6.830
316L	25.05	10.89	33.2372	6.192
Fe-Cu-graphite	24.95	10.38	34.7042	6.838
Fe-Cu-graphite+St.	24.97	9.87	33.3475	6.899

**Table A11:** Δh = 1 mm; F = 250 Kn.

Powder Type	Diameter [mm]	Height [mm]	Mass [g]	Density [g/cm^3^]
316L (40 μm)	24.93	9.54	28.5352	6.127
Fe SC	24.94	9.16	30.2251	6.754
Fe NC	24.94	10.13	34.3349	6.938
316L	25.01	10.83	33.5386	6.303
Fe-Cu-graphite	25.00	10.27	34.5148	6.913
Fe-Cu-graphite+St.	24.95	10.00	33.4947	6.850

**Table A12:** Δh = 1.5 mm; F = 250 kN.

Powder Type	Diameter [mm]	Height [mm]	Mass [g]	Density [g/cm^3^]
316L (40 μm)	24.96	9.62	28.4316	6.040
Fe SC	24.92	9.00	29.8823	6.807
Fe NC	24.94	10.28	35.117	6.992
316L	24.98	10.60	32.9877	6.349
Fe-Cu-graphite	24.92	10.40	34.6018	6.852
Fe-Cu-graphite+St.	24.95	10.05	33.876	6.924

**Table A13:** Δh = 0.5 mm; F = 275 kN.

Powder Type	Diameter [mm]	Height [mm]	Mass [g]	Density [g/cm^3^]
316L (40 μm)	24.94	9.35	28.6961	6.282
Fe SC	24.96	8.63	29.4342	6.970
Fe NC	24.98	10.22	34.9519	6.978
316L	24.97	10.47	33.2073	6.476
Fe-Cu-graphite	24.94	10.06	34.1059	6.939
Fe-Cu-graphite+St.	24.97	9.70	33.0075	6.948

**Table A14:** Δh = 1 mm; F = 275 kN.

Powder Type	Diameter [mm]	Height [mm]	Mass [g]	Density [g/cm^3^]
316L (40 μm)	24.95	9.36	28.3371	6.192
Fe SC	24.93	8.80	29.7639	6.929
Fe NC	25.01	10.17	34.8846	6.982
316L	25.00	10.84	34.1029	6.409
Fe-Cu-graphite	24.96	9.89	33.6758	6.958
Fe-Cu-graphite+St.	24.93	9.83	33.0987	6.965

**Table A15:** Δh = 1.5 mm; F = 275 kN.

Powder Type	Diameter [mm]	Height [mm]	Mass [g]	Density [g/cm^3^]
316L (40 μm)	24.96	9.44	28.7335	6.220
Fe SC	25.00	8.81	29.5322	6.828
Fe NC	24.95	10.26	35.0937	6.996
316L	24.96	10.53	32.7885	6.363
Fe-Cu-graphite	24.97	9.97	34.1299	6.990
Fe-Cu-graphite+St.	24.97	9.60	32.6516	6.975

**Table A16:** Δh = 0.5 mm; F = 300 kN.

Powder Type	Diameter [mm]	Height [mm]	Mass [g]	Density [g/cm^3^]
316L (40 μm)	25.00	9.41	28.8471	6.245
Fe SC	25.00	8.98	30.354	6.886
Fe NC	25.01	9.99	34.043	6.936
316L	25.00	10.69	33.7701	6.435
Fe-Cu-graphite	24.99	10.34	35.4796	6.975
Fe-Cu-graphite+St.	25.00	9.91	33.5024	6.963

**Table A17:** Δh = 1 mm; F = 300 kN.

Powder Type	Diameter [mm]	Height [mm]	Mass [g]	Density [g/cm^3^]
316L (40 μm)	24.97	9.66	29.3859	6.212
Fe SC	24.94	8.95	29.8429	6.825
Fe NC	24.98	10.34	35.0333	6.913
316L	24.97	10.66	33.496	6.416
Fe-Cu-graphite	24.95	10.35	35.1978	6.984
Fe-Cu-graphite+St.	24.97	9.82	32.97	6.974

**Table A18:** Δh = 1.5 mm; F = 300 kN.

Powder Type	Diameter [mm]	Height [mm]	Mass [g]	Density [g/cm^3^]
316L (40 μm)	24.97	9.51	28.7932	6.182
Fe SC	24.96	9.04	30.0739	6.798
Fe NC	25.00	10.17	34.8926	6.989
316L	25.01	10.71	33.8301	6.429
Fe-Cu-graphite	25,00	10.12	34.421	6.993
Fe-Cu-graphite+St.	25.00	9.97	33.7877	6.983

**Table A19:** Δh = 1 mm; F = 325 kN.

Powder Type	Diameter [mm]	Height [mm]	Mass [g]	Density [g/cm^3^]
316L (40 μm)	24.93	9.22	28.6377	6.363
Fe SC	24.92	8.95	30.5463	6.997
Fe NC	24.96	10.18	34.8855	7.003
316L	24.92	10.53	33.5552	6.533
Fe-Cu-graphite	24.92	10.08	34.7429	7.066
Fe-Cu-graphite+St.	24.92	9.67	33.0331	7.003

**Table A20:** Δh = 1.5 mm; F = 325 kN.

Powder Type	Diameter [mm]	Height [mm]	Mass [g]	Density [g/cm^3^]
316L (40 μm)	24.95	9.01	28.3766	6.441
Fe SC	24.94	8.61	29.6346	7.045
Fe NC	24.97	9.95	34.2754	7.034
316L	24.99	10.24	32.9541	6.561
Fe-Cu-graphite	24.99	9.73	34.0094	7.126
Fe-Cu-graphite+St.	24.96	9.83	33.7542	7.017
